# Redox sensing by a Rex-family repressor is involved in the regulation of anaerobic gene expression in *Staphylococcus aureus*

**DOI:** 10.1111/j.1365-2958.2010.07105.x

**Published:** 2010-03-30

**Authors:** Martin Pagels, Stephan Fuchs, Jan Pané-Farré, Christian Kohler, Leonhard Menschner, Michael Hecker, Peter J McNamarra, Mikael C Bauer, Claes von Wachenfeldt, Manuel Liebeke, Michael Lalk, Gunnar Sander, Christof von Eiff, Richard A Proctor, Susanne Engelmann

**Affiliations:** 1 Institute for Microbiology, University of GreifswaldGreifswald, Germany; 2 Department of Medical Microbiology and Immunology, University of WisconsinMadison, Wisconsin, USA; 3 Departments of Biophysical Chemistry, Lund UniversityLund, Sweden; 4 Departments of Biology, Lund UniversityLund, Sweden; 5 Institute for Pharmaceutical Biology, University of GreifswaldGreifswald, Germany; 6 Institute for Medical Microbiology, University Hospital MünsterMünster, Germany

## Abstract

An alignment of upstream regions of anaerobically induced genes in *Staphylococcus aureus* revealed the presence of an inverted repeat, corresponding to Rex binding sites in *Streptomyces coelicolor*. Gel shift experiments of selected upstream regions demonstrated that the redox-sensing regulator Rex of *S. aureus* binds to this inverted repeat. The binding sequence – TTGTGAAW_4_TTCACAA – is highly conserved in *S. aureus*. Rex binding to this sequence leads to the repression of genes located downstream. The binding activity of Rex is enhanced by NAD^+^ while NADH, which competes with NAD^+^ for Rex binding, decreases the activity of Rex. The impact of Rex on global protein synthesis and on the activity of fermentation pathways under aerobic and anaerobic conditions was analysed by using a *rex*-deficient strain. A direct regulatory effect of Rex on the expression of pathways that lead to anaerobic NAD^+^ regeneration, such as lactate, formate and ethanol formation, nitrate respiration, and ATP synthesis, is verified. Rex can be considered a central regulator of anaerobic metabolism in *S. aureus*. Since the activity of lactate dehydrogenase enables *S. aureus* to resist NO stress and thus the innate immune response, our data suggest that deactivation of Rex is a prerequisite for this phenomenon.

## Introduction

*Staphylococcus aureus* is a Gram-positive human pathogen with increasing importance because of its prevalence in hospital settings (Lowy, 1998). Several studies have shown that the virulence of *S. aureus* is determined by the co-ordinated expression of a large variety of virulence factors, which are mainly encoded at highly variable regions of the genome (Foster and Hook, 1998; Dinges *et al*., 2000; Novick, 2003; Foster, 2005). In addition, it has become increasingly accepted that the fitness of this pathogen, which is based on its ability to successfully adapt to host conditions, is also crucial for full virulence (Horsburgh *et al*., 2001a,b; Somerville *et al*., 2003a,b; Kirdis *et al*., 2007; Torres *et al*., 2007; Majerczyk *et al*., 2008). Consequently, elucidation of regulatory mechanisms involved in these processes is required to obtain a comprehensive picture of the pathogenesis of *S. aureus*, and this information may contribute to new therapeutic strategies to treat infections caused by this pathogen.

During the course of an infection *S. aureus* has to cope with varying concentrations of oxygen (Lewis *et al*., 1999). It has been shown previously that *S. aureus* is able to adapt to low oxygen concentrations by the expression of genes involved in nitrate respiration and fermentation. Thus, the loss in NAD^+^ regeneration and ATP synthesis, which follows a switch to a low oxygen environment, is partly compensated by an increase in glycolytic activity and the activation of fermentation pathways. Moreover, transcription of genes possibly involved in secretion of lactate and formate was found to be induced. As a consequence, the fermentation products lactate, formate, ethanol, acetate and 2,3-butanediol accumulate extracellularly. Excessive NADH production is avoided by a decreased synthesis of components of the pyruvate dehydrogenase complex and of the tricarboxylic acid cycle (Fuchs *et al*., 2007) (Fuchs *et al*, unpubl. data).

The regulatory network that enables *S. aureus* to adapt to low oxygen concentration is poorly understood. In response to oxygen depletion, a two-component system, SrrAB (staphylococcal respiratory response AB; synonym SrhSR) that is very similar to ResDE in *Bacillus subtilis,* has been described to be involved in the regulation of fermentation enzymes as well as enzymes belonging to the tricarboxylic acid cycle (Yarwood and Schlievert, 2000; Throup *et al*., 2001; Pragman *et al*., 2004). In line with this, a mutant in *srrAB* is characterized by a severe growth defect under anaerobic conditions (Throup *et al*., 2001). The mechanism that enables the system to modify gene expression in response to varying oxygen concentrations is unknown. Recently, NreABC was described as a specific regulatory system that is essential for transcriptional activation of genes involved in nitrate respiration in *S. aureus* (Schlag *et al*., 2008). NreB is a cytoplasmic histidine sensor kinase. Its sensory domain represents a new type of PAS domains (Taylor *et al*., 1999) containing a [4Fe-4S]^2+^ cluster for sensing oxygen concentration (Mullner *et al*., 2008). In the absence of oxygen, the autokinase activity of NreB is activated and the enzyme self-phosphorylates a conserved histidine residue. The phosphoryl group is then transferred to the response regulator NreC which in turn activates the transcription of its target genes (Kamps *et al*., 2004). A third regulator, ArcR, a member of the Crp/Fnr family of bacterial transcriptional regulators, can bind to a sequence motif similar to Crp binding sites, which is found immediately upstream of a number of anaerobically induced genes. However, using an *arcR* mutant, global gene expression studies showed that only the expression of genes belonging to the arginine deiminase pathway was affected by a loss of this regulator under anaerobic conditions while other fermentation enzymes were unaffected (Makhlin *et al*., 2007). The mechanism by which ArcR is activated under anaerobic conditions remains to be elucidated.

Interestingly, in other bacteria, a similar DNA binding motif as described for ArcR has been identified to be the target of the redox sensing transcriptional regulator Rex. The Rex protein was first described in *Thermus aquaticus* (Du and Pene, 1999). The crystal structure of Rex from *T. aquaticus* (T-Rex) and *Thermus thermophilus* HB8 in complex with NADH and of *B. subtilis* Rex without cofactor has been determined (Du and Pene, 1999; Sickmier *et al*., 2005; Wang *et al*., 2008). Rex is composed of two structural domains: an N-terminal domain that adopts a winged helix–turn–helix fold that most likely interacts with DNA, and a C-terminal NADH binding Rossmann fold domain. Rex is a homodimer stabilized by interaction of the C-terminal α-helices. In the complex with NADH, the N-terminal domains pack close to each other in a compact dimer. This conformation of Rex is unable to bind DNA (Sickmier *et al*., 2005). Rex homologues can be found in a variety of other Gram-positive bacteria such as *Streptomyces coelicolor* (Brekasis and Paget, 2003; Gyan *et al*., 2006).

In the present approach, we have characterized the regulatory role of Rex in anaerobic gene regulation in *S. aureus*. By combining various protein–DNA interaction studies with transcriptional analyses, we have demonstrated that Rex in *S. aureus* acts upon a multitude of anaerobically induced genes, and directly regulates the expression of the ResDE homologue SrrAB. In contrast to the situation in other bacteria, our findings show that in *S. aureus* Rex is a central regulator of adaptation to anaerobic conditions.

## Results

### Characterization of the Rex encoding gene in *S. aureus*

**Fig. 1:**
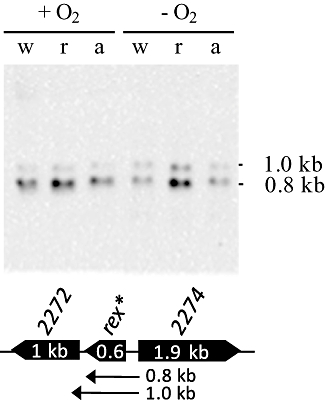
Transcriptional organization of *rex* in *S. aureus*. Total RNA was isolated from SH1000 (w), SH1000 Δ*rex* (r) and SH1000 *ΔarcR* (a) before (+O_2_) and 30 min after imposition of anaerobic conditions (-O_2_). Equal amounts (10 µg) of total RNA were used. The RNA was separated on formaldehyde containing agarose gels and blotted onto positively charged nylon membranes. The membranes were hybridized with the digoxygenin-labelled RNA probes of *rex*. Schematic representation of the gene locus based on the sequence of *S. aureus* NCTC8325 and the transcriptional organization of the predicted operons are shown (‘SAOUHSC_0’ was neglected in the locus tags). The gene used as a probe is marked by an asterisk (*). Methylene blue staining of the membranes is shown in Fig. S2.

To identify whether *S. aureus* contains a Rex-like protein, the complete genome sequence of 13 *S. aureus* strains was screened with a Blast algorithm using the Rex protein of *B. subtilis*. A protein with about 52% identity was found in all *S. aureus* strains. The protein contains 211 amino acids and is encoded by the 636 bp open reading frame SAOUHSC_02273 in *S. aureus* NCTC8325. Transcriptional analyses using *S. aureus* SH1000 as a wild-type revealed two *rex*-specific transcripts of 1.0 and 0.8 kb, as size indicating that the Rex encoding gene is monocistronically transcribed. A slight decrease of the transcript level was observed under anaerobic conditions in the wild-type (Fig. 1). A typical binding motif of Rex as shown for *S. coelicolor* (Brekasis and Paget, 2003) is missing within the *rex* regulatory region. Electrophoretic mobility shift assays (EMSAs) using a DNA fragment extending from 255 bp upstream to 261 bp downstream of the ATG of *rex* showed that Rex does not bind to this region (data not shown). This is in contrast to *S. coelicolor*, where *rex* is preceded by a Rex binding site and the protein has been shown to repress its own transcription (Brekasis and Paget, 2003). However, also in *S. aureus*, the loss of Rex caused a small increase of the *rex* transcript level under both aerobic and anaerobic conditions with a slightly stronger effect under anaerobic conditions (Fig. 1).

### Rex binds to upstream regions of anaerobically induced genes *in vitro*

As shown previously, the alignment of upstream regions of anaerobically induced genes in *S. aureus* revealed the presence of an inverted repeat (TGTGAn_6_TCACA) in front of some of these genes (Fuchs *et al*., 2007). This sequence motif is very similar to Rex binding sites in *S. coelicolor* and, to a lesser degree, to those in *B. subtilis* (Brekasis and Paget, 2003; Schau *et al*., 2004; Larsson *et al*., 2005; Gyan *et al*., 2006). A genome-wide search using the genome sequence of *S. aureus* COL revealed the presence of 461 motifs up to 400 bp upstream of predicted genes: 16 motifs without a mismatch, 25 motifs with one mismatch, and 420 motifs with two mismatches (data not shown).

**Fig. 2:**
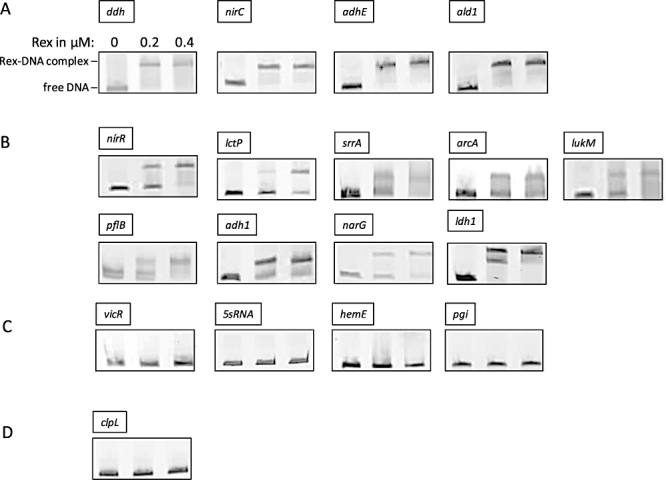
Verification of Rex binding sites by electrophoretic mobility shift assays (EMSAs). Binding of purified Rex to the promoter regions of different genes using EMSAs. The resulting protein–DNA complexes were separated from unbound DNA fragments using native polyacrylamide gels. The DNA fragments were visualized by ethidium bromide staining. Formation of stable Rex–DNA complexes resulted in one or more distinct shifted DNA bands. DNA fragments containing the putative Rex binding site with different Rex binding affinities are shown: (A) strong binding affinity indicated by a complete shift in the presence of 0.2 µM Rex protein; (B) low binding affinity indicated by an incomplete shift in the presence of 0.2 µM Rex protein; (C) no binding in the presence of 0.2 and 0.4 µM Rex protein. (D) As a negative control the upstream region of the anaerobically induced *clpL* gene which lacks a putative Rex binding site was used.

To investigate whether Rex binds in front of these genes, we performed EMSAs using 17 regulatory regions containing the binding motif (note that *ldh1* and *hmp, lctP* and *SACOL2364*, *lukM* and *SACOL2007, ddh* and *frp* might share a possible binding site) (Table 1). With the exception of *ldh1,* where two putative Rex binding sites were identified*,* a single Rex binding site has been predicted in front of these genes. Fourteen of these regulatory regions belong to genes whose expression was shown to be activated under anaerobic conditions. The remaining four regions have been localized in front of genes whose transcription was not affected by oxygen depletion (*vicR, 5sRNA, hemE* and *pgi*), but which possess a binding motif with zero, one or two mismatches to the consensus motif TGTGAn_6_TCACA (Fuchs *et al*., 2007). Based on their binding affinity for Rex, the upstream regions of these genes can be divided into at least three groups. For the first group of upstream regions (i.e. *ddh, nirC, adhE* and *ald1*), a Rex concentration of 0.2 µM led to a complete shift of the used DNA fragments (4 nM) (Fig. 2A). In the second group, by contrast, only incomplete shifts were achieved using the same concentration of Rex (Fig. 2B), suggesting that the protein exhibited a lower affinity for these binding sites. This second group includes upstream regions of the *pflB* operon, *adh1, ldh1,* the *nir* operon, the *arc* operon, the *srrAB* operon, *lukM* and *lctP* genes. For the *ldh1* fragment, two shifted DNA–Rex binding complexes were detected, supporting our hypothesis that two DNA binding sites are present (Fig. 2B). This observation was further supported by performing EMSAs with 28 bp DNA duplexes that contained the different *ldh1* Rex operator sequences (data not shown). For the third group of upstream regions represented by *SACOLSa5SA* (encoding a 5S ribosomal RNA), the *hem* operon, *vicR* and *pgi,* no binding of the purified Rex protein, was observed (Fig. 2C). It is interesting to note that upstream regions of genes whose expression was not induced under anaerobic conditions (i.e. *vicR, 5sRNA, hemE* and *pgi*) (Fuchs *et al*., 2007) were exclusively assigned to the third group. As a negative control, we used upstream regions of anaerobically induced genes that are not preceded by the inverted repeat as shown for *clpL* (Fig. 2D).

**Table 1:** Characterized Rex binding sites in *S. aureus*.

Locus^a^	Gene^a^	Description^a^	Pos^b^	Rex binding motifs^c^	EMSA^d^	mRNA^e^	Prot^e^
SACOL0135	*(adhE)*	Alcohol dehydrogenase, iron-containing	−39		Shift	Up	Up
SACOL0301	*(nirC)*	Formate/nitrite transporter family protein	−51		Shift		
SACOL1478	*ald1*	Alanine dehydrogenase	−38		Shift	Up	
SACOL0660	*(adh1)*	Alcohol dehydrogenase	−306		Shift	Up	Up
SACOL1535	*srrA*	DNA-binding response regulator SrrA	−103		Shift	No change	Up
SACOL2535	*(ddh)*	d-isomer specific 2-hydroxyacid dehydrogenase family protein	−102		Shift		
SACOL2534	*frp*	NAD(P)H-flavin oxidoreductase	−185		[Shift]^f^		
SACOL0204	*pflB*	Formate acetyltransferase	−96		Shift	Up	Up
SACOL0222	*ldh1-1*	l-lactate dehydrogenase	−227		Shift^g^	Up	Up
SACOL0220	*(hmp)*^h^	Flavohemoprotein, putative	−363		[Shift]		
SACOL0222	*ldh1-2*^h^	l-lactate dehydrogenase	−169		Shift^i^	Up	up
SACOL2006	*(lukM)*	Aerolysin/leucocidin family protein	−66		Shift		
SACOL2007		Unknown	−386		[Shift]^j^		
SACOL2363	*(lctP)*	l-lactate permease	−124		Shift	Up	
SACOL2364		Conserved hypothetical protein	−210		[Shift]^k^		
SACOL0019	*yycF (vicR)*	DNA-binding response regulator YycF	−85		Shift^l^	No change	
SACOL0966	*pgi*	Glucose-6-phosphate isomerase	−69		No shift		
SACOL2399	*nirR*	Transcriptional regulator NirR	−64		Shift	Up	
SACOL2657	*arcA*	Arginine deiminase	−79		Shift		
SA2185	*narG*	Respiratory nitrate reductase alpha chain	−107		Shift	Up	
SArRNA04	5sRNA	5S ribosomal RNA	−85		No shift		
SACOL1889	*hemE*	Uroporphyrinogen decarboxylase	−91		No shift		

a Loci, symbols and descriptions are based on TIGR annotation of *S. aureus* COL (http://cmr.jcvi.org). Gene symbols in brackets refer to common annotations found for other *S. aureus* strains.

b Positions of the Rex binding motifs related to the gene start.

c Mismatches to the Rex consensus sequence are underlined.

d Rex binding sites verified by EMSA are indicated by ‘shift’. Motifs showing no Rex binding affinity are indicated by ‘no shift’.

e The influence of Rex on transcript level or protein synthesis. Up = induction in the mutant.

f *ddh* and *frp* share the same binding site.

g EMSA was done with the distal binding site *ldh1-2*.

h *ldh* and *hmp* share the same binding site (*ldh1-2*).

i EMSA was done with the proximal binding site *ldh1-1*.

j *lukM* and SACOL2007 share the same binding site.

k *lctP* and SACOL2364 share the same binding site.

l In EMSA, a distinct shifted band of the regulatory region of *vicR* was only observed in the presence of NAD^+^.

### Rex binding activity is influenced by NADH and NAD^*+*^

**Fig. 3:**
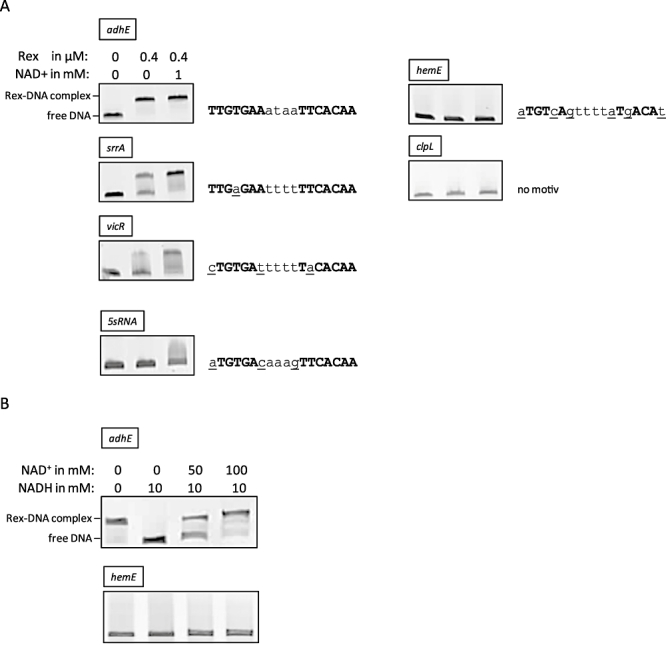
Influence of NAD^+^ and NADH on the Rex affinity to several DNA motifs. A. EMSAs were performed with PCR products of the upstream regions of *adhE, srrA, vicR, 5SrRNA, hemE* and *clpL* after incubation with purified Rex protein in the presence of of NAD^+^. Formation of stable Rex–DNA complexes resulted in a distinct up-shifted DNA band. The respective inverted repeats of the DNA fragments used are shown. Inverted repeats are shown in bold capitals. Mismatches to the consensus sequence are underlined. B. EMSAs were performed with PCR products of the upstream regions of *adhE, nirR, nirC* and *hemE* incubated with 0.4 µM purified Rex protein and different concentrations of NAD^+^ and NADH. Subsequently, the PCR products were separated on native polyacrylamide gels and visualized by ethidium bromide staining.

Because *B. subtilis* Rex is allosterically activated for DNA binding by NAD^+^ (Gyan *et al*., 2006; Wang *et al*., 2008), we analysed the influence of NAD^+^ on DNA binding activity of *S. aureus* Rex to upstream regions of genes with different Rex binding affinities. The addition of NAD^+^ results in an apparent increase in Rex affinity to these upstream regions as shown for *srrAB, vicRK* and *SACOLSa5SA* (Fig. 3A). The same concentration of NAD^+^ did not result in Rex binding to the upstream region of the *hem* operon (Fig. 3A). An influence of NAD^+^ on Rex binding to the *adhE* fragment was seen only when the amount of Rex was decreased to 35 nM (data not shown).

To analyse the influence of the NAD^+^/NADH ratio on DNA binding activity of Rex *in vitro*, we employed EMSAs using the upstream region of *adhE*. As shown in Fig. 3B, the presence of 10 mM of NADH completely prevents the formation of Rex–DNA complexes. This effect is specific for NADH as it was not found for the same concentration of NADPH (data not shown). Rex-binding activity to these DNA fragments was recovered by the addition of increasing amounts of NAD^+^. Thereby, a 5- to 10-fold higher concentration of NAD^+^ compared with NADH was needed to recover DNA binding. These results indicate that NAD^+^ and NADH competitively modulate DNA binding activity of Rex with opposite effects and that Rex binding activity strongly depends on the relative concentrations of NAD^+^ to NADH (Fig. 3B). In these experiments, the upstream region of *hemE* served as a negative control.

**Fig. 4:**
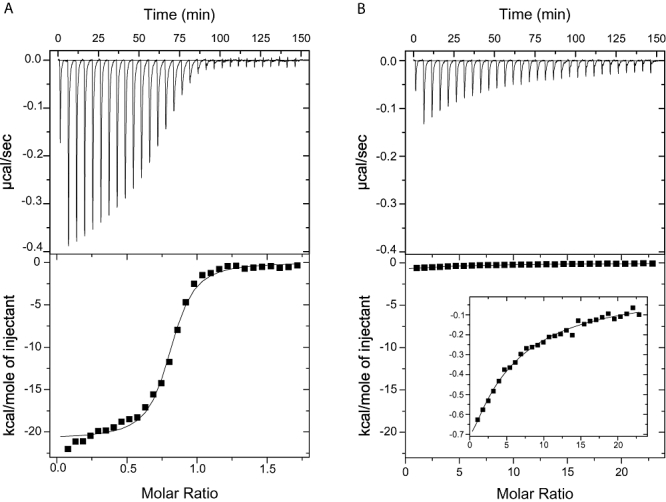
Binding affinity of Rex to NADH and NAD^+^. Isothermal titration calorimetry experiments titrating *S. aureus* Rex with NADH (A) and NAD^+^ (B). Raw data are shown in the top panels and in the lower panels it is fitted using a one-site model. A different scale for the *y*-axis is used in the inserted boxes.

Isothermal titration calorimetry was used to estimate the binding affinities of NADH and NAD^+^ for Rex. Both binding reactions were exothermic. NADH binds with a much higher affinity to Rex, 95 ± 5 nM than NAD^+^, 150 ± 20 µM (Fig. 4). The stoichiometry of the binding reaction with NADH was close to 1 (approximately 0.75). Due to the low affinity for the binding of NAD^+^ the stoichiometry of the reaction could not be unambiguously determined from the experimental data and it was assumed be 1:1.

### Effects of the DNA-target site on Rex–DNA interactions

**Fig. 5:**
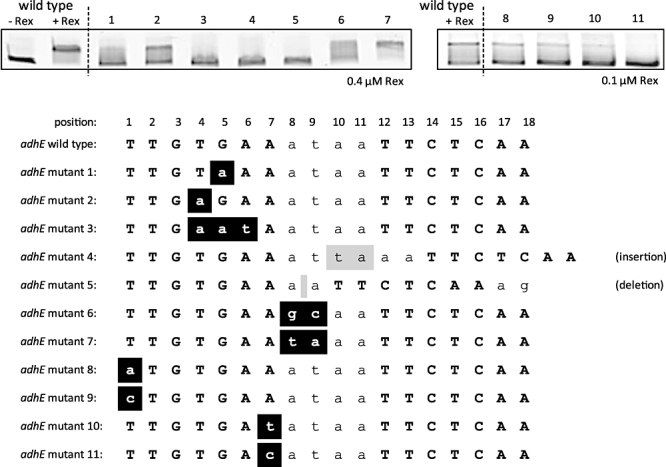
Characterization of the Rex consensus sequence in *S. aureus*. Mutants of the Rex binding site of the *adhE* upstream region were constructed by site-directed mutagenesis. PCR products were incubated with 0.4 or 0.1 µM purified Rex protein. PCR products were run through a native polyacrylamide gel and visualized by ethidium bromide staining. Formation of stable Rex–DNA complexes resulted in a distinct up-shifted DNA band. To illustrate the DNA shift caused by Rex binding, DNA fragments of the wild-type sequence were incubated in the presence (+Rex) and absence (-Rex) of purified Rex protein prior to separation on the gels. The wild-type sequence and the respective mutated binding sequences are shown below. Base pairs that correspond to the wild-type sequence are shown in capital letters. Mutated base pairs are shown in black boxes. Insertions and deletions are shown in grey boxes.

Restriction Endonuclease Protection Assays (REPA) using the *adhE* fragment and purified Rex protein showed that Rex in *S. aureus* exhibits site-directed DNA binding activity to TGTGAn_6_TCACA motifs (data not shown). For a detailed characterization of the DNA binding motif of *S. aureus* Rex, site-directed mutagenesis was performed on the *adhE* upstream region, which showed a complete shift in the presence of 200 nM Rex (Fig. 2A). The binding affinity of Rex was tested for DNA fragments with (i) different base pair substitutions on the distal side of the inverted repeat and (ii) with base pair substitutions or (iii) base pair deletions in the spacer region. As shown in Fig. 5, nucleotide substitutions on the distal side of the inverted repeat at positions 1, 4, 5, 6 and 7 reduced binding activity of Rex to the fragment. Strongest effects were observed when base pairs at position 4 or 5 where exchanged. Among the confirmed Rex binding sites, positions 4 and 5 are highly conserved while mismatches at positions 1 and 7 occur quite frequently (*adh1, ddh, pflB, ldh1-1, ldh1-2, lukM, lctP*) (see also Table 1). Except for *ddh,* these binding motifs belong to those with lower binding affinities (see also Fig. 2). The *srrA* Rex binding sequence is the only sequence with a mismatch at position 4, which still shows Rex binding under *in vitro* conditions (Fig. 2B and Fig. 3A). Exchanges of nucleotides within the spacer regions showed that an optimal spacer has to be A/T rich (see Fig. 5, *adhE* mutant 6 and 7) and might explain the low affinity of Rex to the *SACOLSa5SA* upstream region, which contains several G and C bases within the spacer region (Fig. 3A). Changes in the length of the spacer strongly impact formation of the DNA–Rex complex, suggesting that the distance between the two repeats is important for the geometry of this complex (see Fig. 5, *adhE* mutants 4 and 5).

For the wild-type *adhE* DNA fragment and for the *adhE* DNA fragment of mutant 2 mutated in the distal site of the inverted repeat at position 4 (Fig. 5), we extended the analysis to examine *in vivo* expression in *Escherichia coli* using a β-galactosidase assay. Using the mutated fragment, β-galactosidase expression was de-repressed in the presence of Rex compared with the wild-type DNA fragment, possibly due to a reduced binding affinity of Rex to the mutated Rex binding site (Fig. S1). This confirms our *in vitro* data whereby Rex bound to this DNA fragment with significant lower affinity (Fig. 5).

Additionally, EMSAs were performed using increasing concentrations of Rex (0–1000 nM) to determine the apparent dissociation constant (*K*_d_) of Rex for the upstream regions of *adhE* with no mismatch in the Rex binding site as well as of *adh1* having one mismatch and *nirR* with three mismatches. By this assay, the *K*_d_ of Rex increased in the following order: *adhE* (5.7 × 10^−8^ M), *adh1* (1.2 × 10^−7^ M) and *nirR* (2.7 × 10^−7^ M) (data not shown). These data confirm that mismatches in the inverted repeat reduce the binding affinity of Rex to its target site.

### Colocalization of Rex binding sites and promoter regions

**Fig. 6:**
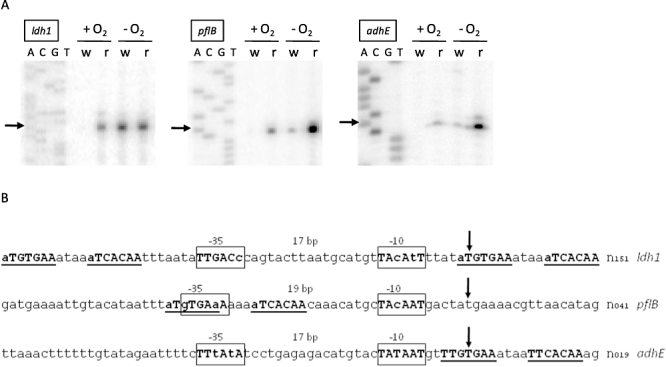
Colocalization of Sigma A consensus sequences and verified Rex binding sites. A. Total RNA was isolated from SH1000 (w) and SH1000 Δ*rex* (r) before and 30 min after a shift to anaerobic conditions. Equal amounts (10 µg) of total RNA were used. The transcription start sites are marked with arrows. Lanes A, C, G and T show the dideoxy sequencing ladders obtained with the equivalent primer. B. The nucleotide sequences around the respective promoter regions of Rex-regulated genes are shown. The transcriptional start points for *ldh1*, *pflBA* and *adhE* obtained by primer extension experiments are indicated by arrows. The potential −10 and −35 regions of SigA-dependent promoters in front of the transcriptional start points of these genes are framed.

We performed primer extension experiments for *ldh1*, *pflB* and *adhE* to determine the transcriptional start sites (Fig. 6A). Total RNA was isolated from *S. aureus* SH1000 and its isogenic *rex* mutant grown in the presence and absence of oxygen. The 5′ ends were mapped to the T located 168 (*ldh1*), 58 (*pflB*) and 36 (*adhE*) base pairs upstream of the coding sequences. The transcriptional start sites are preceded by SigA-like promoter sequences. In each case, the identified Rex binding sites are localized within the promoter regions of these genes or only a few base pairs upstream or downstream of the promoter regions. This supports the idea that binding of Rex at these sites prevents binding of the RNA polymerase and hence hinders transcriptional initiation (Fig. 6B).

### Rex- and ArcR-dependent transcriptional analysis

**Fig. 7:**
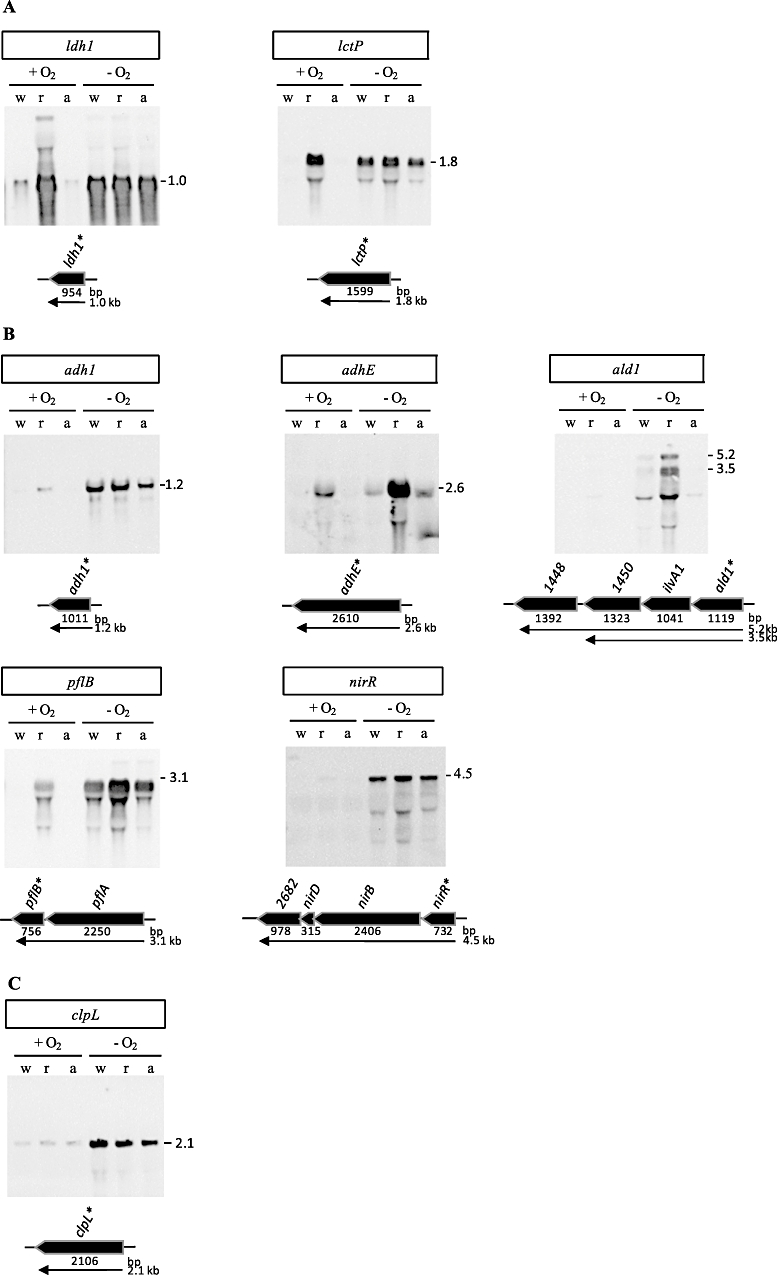
Transcriptional analysis of Rex-regulated genes. Total RNA was isolated from SH1000 (w), SH1000 Δ*rex* (r) and SH1000 *ΔarcR* (a) before (+O_2_) and 30 min after a shift to anaerobic conditions (-O_2_). Ten micrograms of total RNA was used for Northern blot experiments of the genes indicated except for *ldh1* and *pflBA* where 4 µg was used. The RNA was separated on formaldehyde containing agarose gels and blotted onto positively charged nylon membranes. The membranes were hybridized with digoxygenin-labelled RNA probes of the respective genes. Schematic representations of the gene loci based on the sequence of *S. aureus* NCTC8325 and the transcriptional organization of the predicted operons are shown (‘SAOUHSC_0’ was neglected in locus tags). The genes used as a probe are marked by an asterisk (*). Methylene blue staining of the membranes is shown in Fig. S3. Anaerobically induced genes can be divided according to their transcriptional pattern into the following classes: (A) anaerobic induction is solely controlled by Rex; (B) anaerobic induction is partially controlled by Rex; (C) anaerobic induction is not controlled by Rex.

To verify Rex-mediated regulatory effects on transcription of selected genes, we carried out Northern blot experiments with total RNA isolated from the wild-type and the isogenic *rex* mutant under aerobic and anaerobic growth conditions. The kinetic of *ldh1*, *pflBA* and *adhE* transcription supported our results obtained by primer extension analyses. Furthermore, we demonstrated a de-repression of transcription for the majority of operons with verified Rex binding sites under aerobic conditions in the *rex* mutant (Fig. 7A and B). Clearly, there are at least two classes of genes directly regulated by Rex. Class I includes genes whose transcription is upregulated under aerobic conditions in the *rex* mutant and not further induced under anaerobic conditions (Fig. 7A). The second class comprises genes whose transcription is slightly upregulated under aerobic conditions in a Rex-deficient strain but further increased when oxygen is depleted. In these cases, inactivation of Rex is necessary but not sufficient for transcriptional activation and some further mechanisms must also be involved (Fig. 7B). Finally, there are anaerobically induced genes such as *clpL*, whose transcription is not significantly influenced by a mutation in *rex* (Fig. 7C).

Under anaerobic conditions, a higher transcript level of Rex-controlled genes, such as *pflB, ald1 nirR* and *adhE*, was evident in a Rex-deficient mutant compared with the wild-type strain. Most probably, Rex was not completely released from the respective regulatory regions under these conditions in the wild-type. Richardson and co-workers (Richardson *et al*., 2008) have shown that the NAD^+^/NADH ratio of eight is reduced by factor of two after shifting *S. aureus* cells to anaerobic conditions. Thus, for *pflB, ald1, nirR* and *adhE*, an NAD^+^/NADH ratio lower than four might be needed to completely release Rex from the regulatory regions under *in vivo* conditions. This correlates well with our *in vitro* data, which showed the highest binding activities of Rex for *ald1* and *adhE* (Fig. 2A) and that approximately 50% of the *adhE* DNA fragment is released by an NAD^+^/NADH ratio of five (Fig. 3B). On the other hand, transcription of *ldh1* and *lctP* seems to be fully de-repressed under anaerobic conditions in a Rex-proficient background.

Since it has already been shown that the Crp/Fnr-like regulator ArcR binds to the upstream regions of *ldh1*, *ald1*, *lukM*, *arcA* and *srrA in vitro* (Makhlin *et al*., 2007), we analysed its influence on the transcription of these genes. Detailed analyses indicated that ArcR did not significantly influence the transcription of any of these genes neither under aerobic nor under anaerobic conditions (Fig. 7A and B). This is in accordance with already published data, showing that a loss of ArcR only affected the accumulation of proteins belonging to the arginine deiminase pathway (Makhlin *et al*., 2007).

### Protein synthesis profiling to define the Rex modulon

To identify proteins whose synthesis is influenced by Rex, wild-type *S. aureus* SH1000 and its isogenic *rex* mutant were cultivated in synthetic medium to an optical density of 0.5 at 500 nm and then shifted to anaerobic conditions. Thirty and 60 minutes after the shift, protein synthesis profiles were analysed in the presence as well as in the absence of oxygen by *in vivo* labelling of newly synthesized proteins with [^35^S]-methionine. Cytoplasmic proteins were separated on two-dimensional (2D) gels and the protein synthesis patterns obtained were compared. To test whether proteins were differently synthesized, a two-factorial anova involving the oxygen level and the genotype was used (α = 0.01, *P*-values were based on F-distribution).

**Fig. 8:**
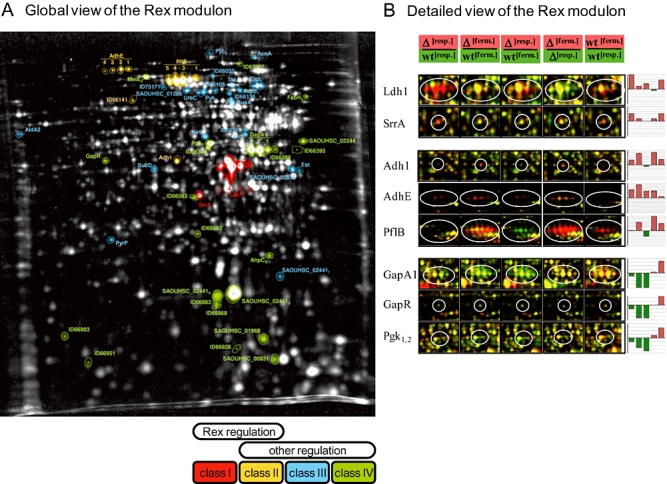
The influence of Rex on anaerobic protein synthesis in *S. aureus*. Cytoplasmic proteins of the wild-type and *rex* mutant were labelled with l-[^35^S] methionine before and 30 and 60 min after a shift to anaerobic conditions and separated on 2D gels. The resulting 2D gel images were analysed using Delta2D software (DECODON, Greifswald). A. Fused 2D image with colour-coded Rex-regulated protein spots of *S. aureus* SH1000. Autoradiograms of the *rex* mutant and the wild-type in the presence and absence of oxygen were combined to generate a fused proteome map and thereby to illustrate the Rex modulon on the 2D image in the standard pH range of 4–7. To show the influence of Rex on the synthesis of proteins, induction ratios of the intensities of each protein spot of the mutant compared with the wild-type were calculated for aerobic as well as for anaerobic conditions. In this way, oxygen-dependent influences on the synthesis of each protein spot were determined. Based on their expression pattern and on the presence of a Rex binding site in front of the respective genes, anaerobically induced or repressed proteins regulated by Rex activity can be divided in the following classes: Class I – anaerobic induction is solely and directly controlled by Rex (red); Class II – anaerobic induction is directly but not solely controlled by Rex (yellow); Class III – synthesis of these proteins is indirectly repressed by Rex (blue); Class IV – synthesis of these proteins is indirectly activated by Rex (green). B. Details of dual channel images of selected proteins whose synthesis was influenced by Rex. The autoradiograms were normalized by using total normalization. The bar graphs on the right display relative synthesis rates (logarithm to the base 2) of the individual proteins at the different time points (red bars = increased synthesis rate, green bars = reduced synthesis rate). SrrA and Ldh1 belong to Class I, Adh1, AdhE and PflB belong to Class II, and GapA1, GapR and Pgk belong to Class IV.

Of the 915 cytoplasmic protein spots detected on the gel, the synthesis of 67 was found to be significantly changed being at least threefold induced or repressed in the presence of Rex (Table 2). Forty-one of these protein spots, which represent 30 proteins, were identified. Taking into consideration both the protein synthesis pattern and the presence of a Rex binding site, Rex-regulated proteins can be divided into at least four classes. As already found by transcriptional analyses, class I and class II include proteins whose genes are preceded by a Rex binding site and the expression of these genes might thus be directly regulated by Rex. These are Ldh1, SrrA, Adh1, AdhE and PflB. The synthesis of class I proteins was upregulated under aerobic conditions in the *rex* mutant and not further induced under anaerobic conditions. A complete de-repression was observed only for Ldh1 and SrrA synthesis in the *rex* mutant under aerobic conditions, indicating that inactivation of Rex might be sufficient to induce expression of the respective genes. In contrast, the synthesis of the second class of Rex-regulated proteins was only slightly affected under aerobic conditions in the absence of Rex but strongly increased when oxygen was limiting. In addition, we found proteins which were obviously influenced by Rex, but for which no Rex binding site was identified in front of their genes. While the synthesis of class III proteins was negatively influenced by Rex, the synthesis of class IV proteins was positively affected by Rex (Fig. 8, Table 2).

**Table 2:** Rex-regulated proteins in *S. aureus*.

				Ratio 30 (60) minutes after oxygen limitation^e^						
Locus (8325)^a^	Protein^b^	ROP^c^	Spot^d^	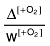	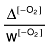	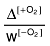	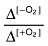	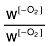	Locus (COL)^f^	Description (COL)^f^
Class I proteins										
SAOUHSC_00206	Ldh1	+	1	13.4 (18.6)	1.5 (2.8)	1.7 (5.5)	0.9 (0.5)	7.7 (3.4)	SACOL0222	l-lactate dehydrogenase
			2	16.2 (12.5)	3.9 (2.5)	6 (6.6)	0.6 (0.4)	2.7 (1.9)	SACOL0222	
			3	3.5 (5.5)	1.3 (1)	2.2 (2.5)	0.6 (0.4)	1.6 (2.2)	SACOL0222	
			4	8.9 (10.3)	2 (1.4)	3.7 (5.9)	0.5 (0.2)	2.4 (1.8)	SACOL0222	
SAOUHSC_01586	SrrA	+		2.9 (3.1)	1.4 (1.2)	1 (1.6)	1.4 (0.8)	2.9 (2)	SACOL1535	DNA-binding response regulator
Class II proteins										
SAOUHSC_00113	AdhE	+	1	13.4 (6.9)	48.1 (12.4)	7.9 (1.8)	6.1 (7.1)	1.7 (4)	SACOL0135	Alcohol dehydrogenase, iron-containing
			2	14.6 (12.3)	67.8 (17.8)	8.9 (2.8)	7.6 (6.4)	1.6 (4.4)	SACOL0135	
			3	3.9 (3.4)	21.2 (5.3)	2.9 (0.6)	7.4 (9.1)	1.4 (5.7)	SACOL0135	
SAOUHSC_00187	PflB	+	1	1 (0.7)	9.2 (3)	0.5 (0.6)	16.9 (5.5)	1.9 (1.3)	SACOL0204	Formate acetyltransferase
			3	1.2 (0.7)	9.3 (3.9)	0.3 (0.2)	28.2 (20)	3.6 (3.7)	SACOL0204	
			4	1.2 (1.1)	7 (3.6)	0.1 (0.1)	72.8 (39.6)	12.4 (11.7)	SACOL0204	
			5	0.9 (0.5)	5.1 (2.6)	0.1 (0.1)	43.2 (43.1)	7.9 (8.1)	SACOL0204	
SAOUHSC_00608	Adh1	+		2.5 (5.2)	9.1 (3.2)	0.8 (0.7)	11 (4.4)	3 (7.2)	SACOL0660	Alcohol dehydrogenase
Class III proteins										
SAOUHSC_00375	GuaA			1 (1.6)	2.4 (4.9)	1.5 (1.9)	1.6 (2.7)	0.6 (0.9)	SACOL0461	GMP synthase/glutamine amidotransferase protein
SAOUHSC_00535	–			1.3 (1.6)	3.3 (2.2)	1.1 (1.5)	2.9 (1.5)	1.2 (1.1)	SACOL0599	Hypothetical protein
SAOUHSC_00909	–			0.8 (0.8)	3.4 (1.4)	0.5 (0.5)	6.6 (3.1)	1.6 (1.7)	SACOL0976	Hydrolase, haloacid dehalogenase-like family
SAOUHSC_01064	Pyc		2	1.4 (11.4)	1.7 (1.4)	5.3 (6.8)	0.3 (0.2)	0.3 (1.7)	SACOL1123	Pyruvate carboxylase
SAOUHSC_01218	SucD			1.4 (1.1)	3.8 (1.5)	1.6 (1.2)	2.4 (1.3)	0.9 (0.9)	SACOL1263	Succinyl-CoA synthetase subunit alpha
SAOUHSC_01266	–			1.3 (1.2)	5.5 (4.4)	6.5 (5.2)	0.8 (0.8)	0.2 (0.2)	SACOL1308	Pyruvate ferredoxin oxidoreductase, alpha subunit
SAOUHSC_01337	Tkt			1.8 (1.8)	2.6 (3.4)	1.9 (2.3)	1.4 (1.4)	0.9 (0.8)	SACOL1377	Transketolase
SAOUHSC_01605	Gnd			1.1 (1.5)	5 (1.7)	1.1 (0.8)	4.7 (2)	1 (1.7)	SACOL1554	6-phosphogluconate dehydrogenase
SAOUHSC_01737	AspS			1.4 (1.6)	2 (3.4)	2.6 (5.6)	0.8 (0.6)	0.5 (0.3)	SACOL1685	Aspartyl-tRNA synthetase
SAOUHSC_01839	TyrS			1 (1.4)	1.3 (3.2)	0.8 (1.8)	1.6 (1.8)	1.3 (0.8)	SACOL1778	Tyrosyl-tRNA synthetase
SAOUHSC_02142	AldA2			4.5 (1.1)	0.5 (0.8)	1.1 (1.1)	0.4 (0.7)	4 (1)	SACOL1984	Aldehyde dehydrogenase
SAOUHSC_02441	Asp23		1	0.8 (0.6)	3 (2.5)	0.9 (0.8)	3.4 (3.3)	0.9 (0.8)	SACOL2173	Alkaline shock protein 23
SAOUHSC_02561	UreC			1.9 (1.4)	2.3 (3)	2 (1.9)	1.2 (1.6)	1 (0.7)	SACOL2282	Urease subunit alpha
SAOUHSC_02862	ClpL			1.5 (1.3)	2.9 (4.1)	0.6 (0.3)	5.2 (12.8)	2.6 (4)	SACOL2563	ATP-dependent Clp protease, putative
Class IV proteins										
SAOUHSC_00338	MetE		2	1.6 (1.4)	0.3 (0.3)	1 (1.3)	0.3 (0.2)	1.5 (1.1)	SACOL0428	5-methyltetrahydropteroyltriglutamate-homocysteine methyltransferase
SAOUHSC_00365	AhpC		3	0.2 (0.8)	0.4 (0.6)	0.8 (1.1)	0.6 (0.6)	0.2 (0.8)	SACOL0452	Alkyl hydroperoxide reductase, C subunit
SAOUHSC_00794	GapR			0.7 (1)	0.3 (0.6)	0.3 (0.3)	1 (2.2)	2.8 (3.8)	SACOL0837	Gap transcriptional regulator
SAOUHSC_00795	GapA1		1	0.6 (0.7)	0.3 (0.6)	0.3 (0.1)	1.1 (5.1)	2.2 (5.7)	SACOL0838	Glyceraldehyde 3-phosphate dehydrogenase
			2	0.7 (1.3)	0.2 (0.7)	0.2 (0.2)	1.1 (4.1)	3.4 (7.4)		
			3	1 (0.6)	0.3 (0.6)	0.3 (0.1)	1.1 (5.4)	3.6 (5.3)		
SAOUHSC_00796	Pgk		2	0.8 (1)	0.3 (0.9)	0.2 (0.2)	1.3 (4.7)	3.6 (5.4)	SACOL0839	Phosphoglycerate kinase
SAOUHSC_00831	–			0.6 (0.6)	0.2 (0.7)	0.4 (0.4)	0.6 (1.5)	1.3 (1.2)	SACOL0872	OsmC/Ohr family protein
SAOUHSC_00920	FabH		3	1.4 (0.2)	0.4 (0.3)	1.3 (0.7)	0.3 (0.5)	1 (0.3)	SACOL0987	3-oxoacyl-(acyl carrier protein) synthase III
SAOUHSC_01347	–			0.2 (0.4)	1.4 (0.6)	0.5 (0.9)	2.7 (0.6)	0.4 (0.4)	SACOL1385	Aconitate hydratase
SAOUHSC_01968	–			0.3 (0.3)	0.3 (0.5)	0.4 (0.3)	0.9 (1.5)	0.9 (0.9)	SACOL1894	HIT family protein
SAOUHSC_02244	–			0.6 (0.6)	0.3 (0.4)	0.7 (0.4)	0.4 (1)	0.9 (1.4)	–	
SAOUHSC_02441	Asp23		3	0.7 (0.8)	0.3 (0.5)	0.6 (0.6)	0.6 (1)	1.2 (1.4)	SACOL2173	Alkaline shock protein 23
SAOUHSC_02441	Asp23		4	0.5 (0.5)	0.2 (0.6)	0.3 (0.3)	0.5 (1.8)	1.3 (1.6)		

a Loci are based on TIGR annotation of *S. aureus* COL (http://cmr.jcvi.org).

b Symbols are based on TIGR annotation of *S. aureus* COL or N315 (italic) using Geneplot of NCBI (http://www.ncbi.nlm.nih.gov).

c The presence of a Rex binding site (ROP) in front of the respective genes indicated by +.

d Spots representing the same protein are distinguished by an additional spot number.

e Protein spots whose synthesis was significantly induced or repressed at least threefold in the mutant compared with the wild-type.

f Loci and product descriptions are based on TIGR annotation of *S. aureus* COL using Geneplot of NCBI.

### The influence of Rex on extracellular metabolites of *S. aureus*

As we have shown, Rex is involved in the regulation of metabolic pathways that mediate NAD^+^ regeneration and ATP synthesis, such as lactate, ethanol and formate formation, nitrate and nitrite respiration, the arginine deiminase pathway and of transporters that might extrude fermentation products (i.e. lactate and formate). Deeper insights into the physiological consequences of this adaptive process can be obtained by determining whether or not the proteins accumulating under certain conditions are active. An analysis of extracellular metabolites can be the first step in this approach.

**Fig. 9:**
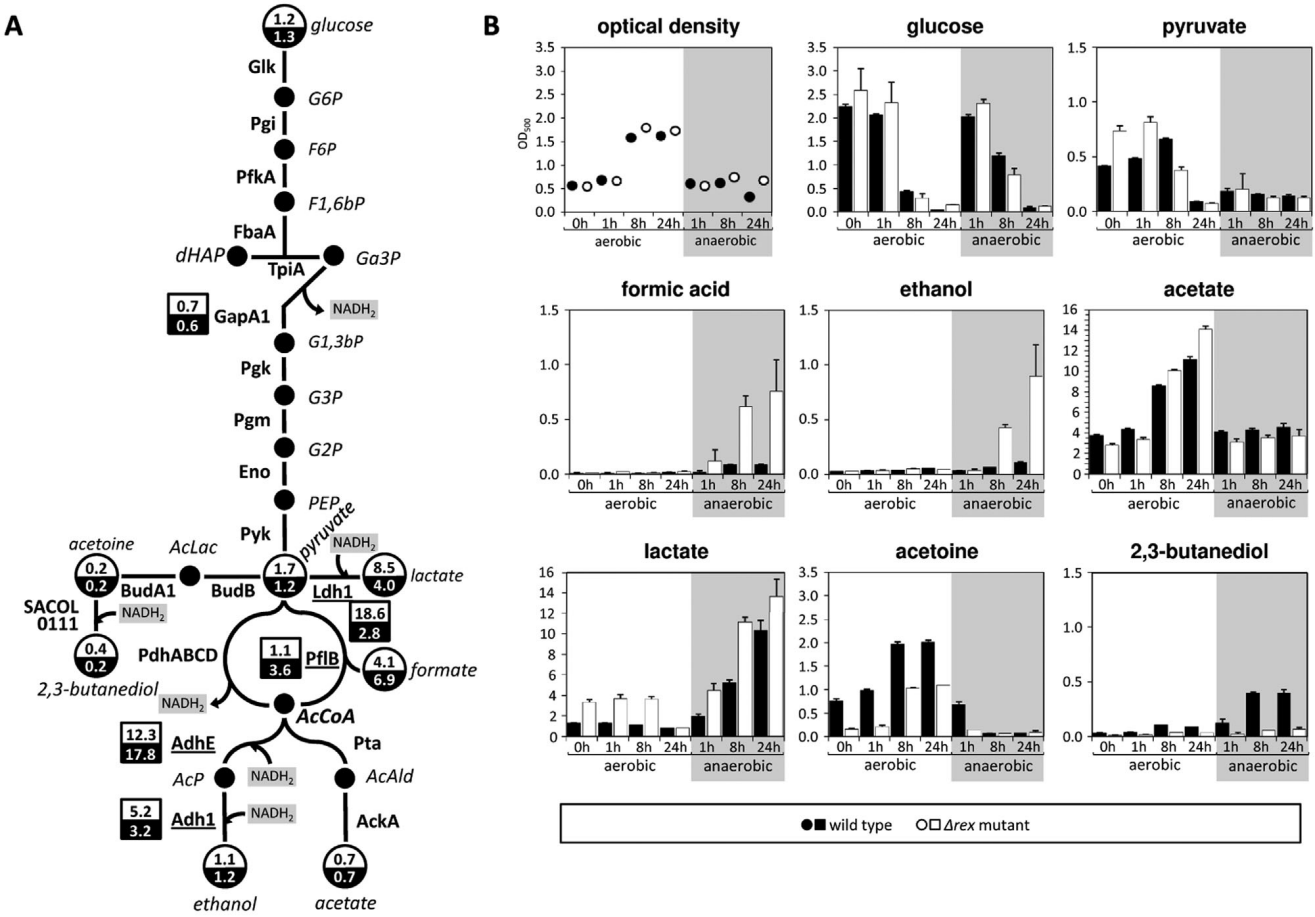
Impact of Rex on selected metabolic pathways. A. Selected proteomic and metabolomic data of the glycolysis and fermentation pathways. Protein synthesis rates (*rex* mutant/wild-type) of selected enzymes are shown in squares and ratios of selected metabolites (*rex* mutant/wild-type) are shown in circles for aerobic (white) and anaerobic (black) conditions. Proteins whose genes are directly Rex-regulated are underlined. Cells were grown in synthetic medium without MOPS buffer to an optical density of 0.5 and shifted to anaerobic conditions. Cytoplasmic proteins of the wild-type and *rex* mutant were labelled with l-[^35^S]-methionine before and 60 min after a shift to anaerobic conditions and separated on 2D gels. The resulting 2D gel images were analysed using Delta2D software (DECODON, Greifswald). For metabolite analyses, samples were taken from the aerobically grown culture and from the anaerobically grown culture immediately before (only aerobic culture) and 1, 8 and 24 h after shifting to anaerobic conditions. Cells were separated from the supernatant by filtration and the supernatants obtained were used for further analyses. B. Extracellular metabolite analyses by ^1^H-NMR. Glucose, pyruvate, formic acid, ethanol, acetate, lactate, acetoine and 2,3-butanediol were detected and quantified by ^1^H-NMR. The graphs show the increase of the concentration in mM.

We analysed the extracellular metabolites of the wild-type and the *rex* mutant under aerobic and under anaerobic conditions (Fig. 9). Glucose was used as the main carbon source by both strains under aerobic and anaerobic conditions. At glucose excess, there was a high secretion of acetate and pyruvate under aerobic conditions indicating overflow metabolism. Moreover, acetoine has been detected as a further overflow metabolite but in significantly lower amounts in the *rex* mutant. Interestingly, while pyruvate is reused when glucose is fully consumed, acetate and acetoine remained in the supernatant. Lactate was produced under aerobic conditions in higher amounts in the mutant strain. After shifting the cells to anaerobic conditions, comparable amounts of lactate were produced as the major end-product for both strains. Formic acid and ethanol were found in significantly higher amounts in the mutant. In contrast, formation of 2,3-butanediol was impaired in the mutant (Fig. 9), which is possibly caused by a reduced acetoine production compared with the wild-type.

## Discussion

Since oxygen is indispensable for an operative aerobic respiratory chain in *S. aureus*, anaerobic adaptation is mainly focused on achieving a balanced intracellular NAD^+^/NADH ratio as well as a balanced ATP pool (Fuchs *et al*., 2007). The same is true when the function of the respiratory chain is blocked (e.g. by mutations in *hemB* or *menD* or by nitric oxide) (Kohler *et al*., 2003; 2008; Hochgräfe *et al*., 2008). In *S. aureus*, a drop in the intracellular NAD^+^/NADH ratio most likely leads to inactivation of Rex, and consequently to a de-repression of Rex-regulated genes. The Rex regulon has been defined by EMSAs, Northern analysis and proteomics. Nineteen of the 29 genes/proteins postulated to be directly regulated by Rex have been confirmed by these means. At least two classes of genes defined by their expression patterns are directly regulated by Rex. Genes belonging to the first class showed maximal expression in a *rex* mutant already under aerobic conditions. Rex-associated de-repression might be the major regulatory mechanism for the expression of these genes. This group includes genes encoding lactate dehydrogenase and lactate permease. This suggests that in *S. aureus* NAD^+^ regeneration by lactate fermentation represents an immediate response to a drop in the NAD^+^ pool. In contrast, transcription of the second group of genes was only marginally affected under aerobic conditions in the absence of Rex and was only strongly upregulated when oxygen becomes limiting. Among these genes are those encoding enzymes involved in formate and ethanol formation, in nitrate/nitrite respiration and the alanine dehydrogenase. We therefore postulate the existence of one or more accessory regulators that might be activated under oxygen limitation. In fact, neither conserved SigA- nor SigB-dependent promoter consensus sequences have been found in the upstream regions of *narG*, *nirR* and *arcA*, which were also shown to be positively regulated by NreB, NirR and/or ArcR (Neubauer *et al*., 1999; Fedtke *et al*., 2002; Kamps *et al*., 2004; Makhlin *et al*., 2007; Schlag *et al*., 2008). Moreover, the expression of *pflB*, *adhE*, *adh1*, *nirR* and *ald1* is additionally regulated by a menadione-dependent regulatory mechanism possibly mediated by SrrAB, whose activity might be induced under anaerobic conditions (Kohler *et al*., 2008).

A more detailed characterization of Rex binding affinities to the DNA binding motif of the *adhE* upstream region (Fig. 5) indicates that the 18-base-pair motif TTGTGAAW_4_TTCACAA is the Rex consensus sequence in *S. aureus*. The genome-wide search for Rex binding sites in *S. aureus* COL was improved by using this more specific consensus sequence for Rex binding. Allowing up to three mismatches, 54 potential Rex binding sites were identified (Table S1, note that some of the binding motifs may be shared by divergently oriented genes). Twenty-two of the downstream located genes are among genes whose expression was upregulated under anaerobic conditions (Fuchs *et al*., 2007). For 14 of these binding sites, we verified Rex binding by EMSAs (Table 1, Table S1). As expected, binding sites with high binding affinity to Rex (i.e. *adhE*, *ald1* and *nirC*) exhibit no deviations from the consensus sequence while those with lower binding affinity show at least one mismatch (e.g. *nirR, pflB, adh1, lukM, lctP, ldh1*). The inverted repeat of the *hem* operon that is not targeted by Rex under *in vitro* conditions failed the more stringent search criteria.

In addition to the genes/proteins whose expression might be directly regulated by Rex, we identified 25 proteins whose synthesis was significantly changed in the presence of Rex, but for which no Rex binding site was found in front of their genes. Indirect effects of Rex might occur via additional regulators that are directly regulated by Rex such as SrrA and NirR or via changes in the metabolism that in turn can affect gene expression. Remarkably, in the present approach Rex has been identified as a negative effector of SrrA synthesis while the influence of Rex on the transcript level of *srrAB* was negligible (data not shown).

In addition to a deeper understanding of the physiological behaviour of the facultative anaerobe *S. aureus*, the study also provides first insights into the role of the redox sensing regulator Rex in *S. aureus* virulence. Recently, upregulation of Ldh1 and Ddh under NO stress has been described (Richardson *et al*., 2006; Hochgräfe *et al*., 2008). NO is generated by the NO synthase in activated phagocytes in the human host and plays a crucial role in innate immunity (Fang, 2004). Respiratory terminal oxidases, the nitrate reductase and the pyruvate formate lyase as the first step of ethanol fermentation are targets for destructive NO (Brown *et al*., 1997; Stamler *et al*., 2001). Disabling of these three pathways for NAD^+^ regeneration may be countered by upregulation of d- and l-lactate production as a key factor in aerobic and anaerobic NO resistance. Since it has been shown that the activity of lactate dehydrogenase enables *S. aureus* to resist the innate immune response (Richardson *et al*., 2008), our finding suggest that deactivation of Rex might be crucial for this phenomenon and hence that Rex has a crucial role to play in NO resistance and thus in virulence of *S. aureus*.

The redox sensing protein Rex is highly conserved among Gram-positive bacteria (Brekasis and Paget, 2003). Searching for Rex and potential Rex binding sites (TTGTGAAW_4_TTCACAA) in different staphylococcal species (*S. aureus*, *Staphylococcus epidermidis*, *Staphylococcus saprophyticus* and *Staphylococcus haemolyticus*) shows that although all staphylococci possess a Rex encoding gene the direct overlap between the lists of potentially Rex-regulated genes is rather small. Only four genes are preceded by a potential Rex binding site in all four species: *lctP* and the divergently transcribed locus SACOL2364 (hypothetical protein), SACOL2491 (hypothetical protein) and the *srrAB* operon. Rex-dependent regulation of lactate production appeared to be also highly conserved. At least one of the two anaerobic lactate dehydrogenases (*ldh* or *ddh*) was identified as a potential Rex target in all four species. Overall the conservation pattern of the putative Rex regulons showed a mosaic-like structure. For instance, *adhE* and *pflB* are confined to *S. aureus* and *S. epidermidis*. Furthermore, *adh1,* although highly conserved in *S. aureus* and *S. epidermidis* (SERP0257), is only preceded by a Rex binding site in *S. aureus*. Moreover, dissimilatory nitrate and nitrite reductase activity, nitric oxide dioxygenase Hmp and the *arc* operon may be subjected to Rex control in some but not all of these species (data not shown).

In *B. subtilis* and *S. coelicolor*, Rex regulates genes encoding components of the respiratory chain (e.g. cytochrome *bd* terminal oxidases, haem biosynthesis) and NADH dehydrogenases. Most interestingly, cytochrome *bd* has a high oxygen affinity and might thus enable bacteria to use oxygen for oxidative phosphorylation and NAD^+^ regeneration under conditions of low oxygen tension (Brekasis and Paget, 2003; Schau *et al*., 2004; Larsson *et al*., 2005; Gyan *et al*., 2006; Puri-taneja *et al*., 2007; Wang *et al*., 2008). Anaerobic metabolic pathways are mainly regulated by other regulatory systems such as Fnr and ResDE (Cruz Ramos *et al*., 1995; Nakano *et al*., 1996; Reents *et al*., 2006). This is in contrast to *S. aureus* where in general genes involved in anaerobic respiration and fermentation are under the control of the redox sensitive regulator Rex, while genes associated with haem and cytochrome biosynthesis might not be subject to Rex-dependent regulation. For example, in front of the *hem* operon which has been shown to be under direct regulation of Rex in *S. coelicolor* (Brekasis and Paget, 2003), a degenerate Rex binding site has been identified in *S. aureus* but neither binding of Rex to this region nor Rex-dependent transcription of the operon has been observed. In contrast, Rex-mediated transcriptional control of genes that contribute to anaerobic metabolism and thereby to oxidation of NADH is apparent for *S. aureus* and may represent a common theme of the Rex regulons among the staphylococcal species. In conclusion, this variation between the regulons in the different staphylococcal species, *B. subtilis* and *S. coelicolor* may reflect individual adaptations of their aerobic and anaerobic metabolism in response to the fluctuating redox environments imposed by the ecological niches they inhabit.

## Experimental procedures

### Bacterial strains, mutant construction and growth conditions

*Staphylococcus aureus* strains used in the present study are shown in Table 3. For construction of *S. aureus*Δ*rex* (AK1), we used a well-developed methodology for allelic exchange in conjunction with a endonuclease-based counter selection against allelic exchange vector-containing strains to create a *rex* null mutant of *S. aureus* strain SH1000 (Janzon and Arvidson, 1990; Posfai *et al*., 1999). Allelic exchange used plasmid pAK001. This plasmid is a derivative of pJM930, an *E. coli–S. aureus* shuttle vector that in *S. aureus* is temperature sensitive for replication and confers chloramphenicol resistance. In addition, pJM930 carries a 30 bp recognition site for the I-SceI homing endonuclease. Plasmid pAK001 contains a mutant allele of *S. aureus rex* (Δ*rex*_3–22_) on a 1788 bp KpnI DNA fragment. Δ*rex*_3–22_ was created using three rounds of PCR. In the first set of reactions, *S. aureus* strain SH1000 chromosomal DNA was used to amplify either 0.7 or 1.0 kb fragments using primers outside of *rex* and mutagenic primers internal to *rex*. The amplified products were annealed, and the mutant fragment was amplified using the primers outside of *rex*. The mutation in *rex* consists of a 20 bp deletion at positions 3 through 22 relative to the translational start site of wild-type *rex*. Plasmid pAK001 was electroporated into *S. aureus* strain RN4220, and subsequently into SH1000 as described previously (Kraemer and Iandolo, 1990). Selection for pAK001 used chloramphenicol at the permissive temperature for independent replication of the allelic exchange vector. Plasmid integrates were selected at 42°C, the non-permissive replication temperature, in media containing chloramphenicol. Mutants were obtained using counter-selection against plasmid-integrates and cytoplasmic plasmids by transduction of erythromycin resistance on plasmid pJM928, which constitutively produces the nuclease I-SceI. *S. aureus* containing wild-type *rex* were distinguished from those carrying the Δ*rex*_3–22_ mutation by PCR. PCR was performed using primers that amplified a 297 bp fragment of *rex*, but not Δ*rex*_3–22_ DNA (the primer included 3′ sequence that corresponds to the deleted bases in Δ*rex*_3–22_). The Δ*rex*_3–22_ ORF was amplified from a candidate mutant strain and the DNA sequence of the amplified product was determined and compared with the *rex* sequence from SH1000. In Western blot experiments using polyclonal antibodies against *S. aureus* Rex, no Rex protein could be detected in the *rex* mutant (data not shown).

**Table 3:** Strains and plasmids used in this study.

Strains or plasmids	Description	Source or reference
Strains		
*Escherichia coli*		
*DH5α*	*supE44*Δ*lacU169* (f80*lacZ*DM15) *hsdR17 recA1 endA1 gyrA96 thi-1 relA1*	Hanahan (1983)
*Staphylococcus aureus*		
RN4220	Restriction negative strain	Kreiswirth *et al*. (1983)
SH1000	Functional *rsbU* strain derived from *S. aureus* 8325-4	Horsburgh *et al*. (2002)
SH1000 Δ*rex* (AK1)	SH1000 with a 20 bp deletion in *rex*	This study
SH1000 Δ*arcR*	SH1000 with a deleted *arcR* gene	Makhlin *et al*. (2007)
Plasmids		
pJM930	pBT2 based vector containing a I-SceI restriction site	This study
pAK001	pJM930 with mutated *rex* gene for recombination	This study
pJM928	pSPT181 shuttle vector, constitutively expressing I-SceI	Heinrichs *et al*. (1996)

For cultivation experiments, cells were first grown under aerobic conditions either in 100 ml Lennox L Broth (LB Broth) (Invitrogen, Karlsruhe, Germany) for transcriptional studies or in 100 ml synthetic medium for proteomics experiments using 500 ml Erlenmeyer flasks, which were vigorously agitated at 37°C to an optical density (OD_500_) of 0.5. One part of the culture was shifted to anaerobic growth by transferring 50 ml of the culture to Falcon tubes, which were completely filled with bacterial cell culture and incubated under vigorous agitation at 37°C. The remaining part of the culture was further cultivated under aerobic conditions. Anaerobic conditions were verified by using 0.001% resazurin as a redox indicator (Maeda *et al*., 2001; Fuchs *et al*., 2007). Cells grown under aerobic or anaerobic conditions were harvested 30 and 60 min after the time point of the shift.

### Purification of recombinant Rex protein

The *rex* gene of *S. aureus* N315 (SA1851) was amplified with synthetic oligonucleotides (Table S2) by PCR, and cloned into pPR-IBA1 (IBA, Göttingen, Germany), following the manufacturer's instructions. *E. coli* DH5α (Invitrogen) was used for plasmid amplification. The insert was confirmed by sequencing (4base lab GmbH, Reutlingen, Germany). The recombinant C-terminal *Strep*-tagII tagged Rex protein was purified from *E. coli* BL21 pLysS (Invitrogen), using a *Strep*-tag purification column (IBA), according to the manufacturer's protocol.

The absorbance at 340 nm of the purified Rex protein was measured using *nanodrop DN-1000* (Agilent, Böblingen, Germany) and *ultrospec 3100 pro* (Amersham biosciences, Freiburg, Germany). No absorbance peak at 340 nm, which is characteristic for NADH, was detected (data not shown) unlike the case for T-Rex, where stoichiometric levels of NADH co-purified with the protein (Sickmier *et al*., 2005).

### Protein–DNA interaction studies

For EMSAs, the upstream regions of several genes were amplified by PCR with synthetic oligonucleotides (Table S2). Purified PCR products (4 nM) were incubated with different amounts of purified Rex and different amounts of sheared salmon testis DNA in reaction buffer [20 mM Tris-HCl pH 7.5, 2 mM EDTA, 3% (w/v) Ficoll 400, 0.5 mM DTT, 150 mM NaCl] for 15 min at room temperature. The reaction mixture was analysed in a 5.4% acrylamide/bisacrylamide gel under native conditions. DNA fragments were visualized by ethidium bromide staining. To confirm specific protein DNA interactions, replicates were carried out with 1 and 5 µg sheared salmon testis DNA. A protein DNA interaction was judged to be specific, when the shifted band remained stable in the presence of a fivefold excess of sheared salmon testis DNA. Each determination was repeated at least one time with PCR products obtained from an independent PCR.

For analysing the influence of NAD^+^ and NADH on the binding affinity of Rex, EMSAs were performed as described above with the following modifications: The reaction mixture was supplemented with NAD^+^ and/or NADH in different amounts and the Tris-HCl buffer was replaced by HEPES buffer to avoid DNA-nicotinamide co-precipitation. Parallel reactions were carried out in the presence of 0.4 µM Rex protein.

### Isothermal titration calorimetry

Isothermal titration calorimetry experiments were performed by titrating a nucleotide solution containing either 75 µM NADH or 1 mM NAD^+^ into a solution with 10 µM Rex. UV-visible spectrophotometric analysis of the preparation of Rex used for the measurements showed that the sample did not contain NADH. Both titrations were performed in replicates. All experiments were performed in 100 mM potassium phosphate buffer, pH 7.5 at 25°C using a VP-ITC microcalorimeter (MicroCal, Northampton, MA, USA). The reaction cell contained 1.42 ml of protein in buffer and the reference cell contained distilled H_2_O. After a first injection of 5 µl 29 injections of 10 µl were made with 5 min spacing between the injections. Data were fitted with the ORIGIN 7 software (MicroCal) using a one-site model. The stoichiometry (n) of the binding reaction was set to 1 for the titration with NAD^+^.

### Site-directed mutagenesis of the *adhE* upstream region

The *adhE* upstream region was amplified by PCR with synthetic oligonucleotides (Table S2), and cloned into pRSETA (Invitrogen), using XhoI and PvuI. *E. coli* DH5α (Invitrogen) was used for plasmid amplification. Site-directed mutagenesis was performed with the GeneTailor site-directed mutagenesis kit (Invitrogen), according to the manufacturer's protocol. The mutated inserts were confirmed by sequencing (4base lab GmbH), and analysed in EMSA.

### Transcription analyses

For Northern blot analyses, total RNA was isolated according to a modified acid phenol method (Gertz *et al*., 1999; Fuchs *et al*., 2007). Northern blot analyses were performed according to the protocol published by (Wetzstein *et al*., 1992). To prepare Dig-labelled RNA probes, synthetic oligonucleotides (Table S2) were used for PCR. Purified PCR products were used as templates for T7 RNA polymerase mediated *in vitro* transcription with a NTP labelling mix (Roche Diagnostics, Mannheim, Germany). Chemiluminescent signals were detected with a Lumi-Imager and visualized with Lumi-Analyst (Roche Diagnostics). Experiments were carried out in duplicate.

For primer extension analyses, synthetic oligonucleotides (Table S2) were labelled with [γ-^32^P]-ATP. For sequencing, upstream regions were amplified with synthetic oligonucleotides (Table S2) by PCR and used as templates. Sequencing was performed according to the method of (Sanger *et al*., 1977). Five micrograms of isolated total RNA was applied for reverse transcription with *SuperScriptII* reverse transcriptase (Invitrogen). Sequencing reactions and reverse transcription reactions were analysed on polyacrylamide gels, and visualized on a phosphor storage screen with a typhoon scanner (Amersham Biosciences). Experiments were carried out in duplicate.

### Preparation of pulse labelled protein extracts

For the analysis of protein synthesis under aerobic and anaerobic growth conditions the proteins were pulse labelled with l-[^35^S]-methionine. Pulse labelling was performed for aerobically and anaerobically grown cells 30 and 60 min after the shift as described previously (Fuchs *et al*., 2007).

Protein extracts from these cells were prepared by re-suspension of the cell pellet in 400 µl TE containing 0.01 mg lysostaphin followed by incubation on ice for 10 min and disruption by sonication. The cell lysate was cleared by a first centrifugation step (9000 *g*, 10 min, 4°C) in order to remove cell debris followed by a second centrifugation step (21 000 *g*, 30 min, 4°C) to remove insoluble and aggregated proteins, which could interfere with isoelectric focusing. The extent of radiolabel incorporation into proteins was determined by standard procedures (Bernhardt *et al*., 1999). The protein concentration was determined using the Roti Nanoquant reagent (Roth, Karlsruhe, Germany) and protein solutions were stored at −20°C.

### Preparation of cytoplasmatic proteins for preparative 2D gel electrophoresis

Cells of 50 ml culture were harvested on ice and centrifuged for 10 min at 7000 *g* and 4°C. Cells were washed twice with ice-cold TE buffer and resuspended in 1 ml of lysis buffer (10 mM Tris, 1 mM EDTA, 1 mM phenylmethylsulphonyl fluoride, pH 7.5). For mechanical disruption, the cell suspension was transferred to screw-cap microtubes (Sarstedt, Nümbrecht, Germany) containing 500 µl of glass beads (diameter 0.10–0.11 mm, Sartorius, Göttingen, Germany). Cells were disrupted by homogenization using a Ribolyser (Peqlab, Erlangen, Germany) at 6.5 m/s for 35 seconds. The lysate was centrifuged for 25 min at 21 000 *g* (4°C). In order to remove membrane fragments and insoluble proteins, the centrifugation step was repeated for 45 min at 21 000 *g* (4°C). The protein concentration was determined using Roti-Nanoquant (Roth), and the protein solution was stored at −20°C.

### Analytical and preparative two-dimensional polyacrylamide gel electrophoresis (PAGE)

Two-dimensional PAGE was performed using the immobilized pH gradient (IPG) technique described previously (Büttner *et al*., 2001). In the first dimension, the protein samples were separated on IPG strips (GE-Healthcare, Little Chalfont, UK) in the pH range of 4–7. For analytical 2D PAGE 100 µg of radioactively labelled protein extracts was loaded onto the IPG strips. The 2D gels were stained with Sypro Ruby (Invitrogen, Eugene, OR, USA) according the recommendations of the manufacturer. The stained gels were scanned with the Typhoon 9400 Variable Mode Imager (Amersham Biosciences). Afterwards the gels were dried on a vacuum dryer onto Whatman paper. The dried gels were exposed to storage phosphor screens (Molecular Dynamics, Krefeld, Germany) and scanned with the Typhoon 9400 Variable Mode Imager. The exposure time of each gel depended on the signal intensity.

For identification of proteins by mass spectrometry preparative 2D PAGE was performed. Some 200 or 250 µg of protein extracts was loaded onto the IPG strips (GE-Healthcare) in the pH range of 4–7. The resulting 2D gels were fixed as described above and stained with Sypro Ruby. The stained gels were scanned with the Typhoon 9400 Variable Mode Imager (Amersham Biosciences).

### Protein identification by mass spectrometry

For identification of proteins by MALDI-TOF-MS, Sypro Ruby stained protein spots were cut from gels using a spot cutter (Proteome WorkTM) with a picker head of 2 mm and transferred into 96-well microtiter plates. Digestion with trypsin and subsequent spotting of peptide solutions onto the MALDI targets were performed automatically in the Ettan Spot Handling Workstation (GE-Healthcare) using a modified standard protocol (Eymann *et al*., 2004). MALDI-TOF-MS analyses of spotted peptide solutions were carried out on a Proteome-Analyzer 4700 (Applied Biosystems, Foster City, CA, USA). The spectra were recorded in a reflector mode in a mass range from 900 to 3700 Da. Automatic or manual calibration was performed as described previously (Eymann *et al*., 2004). After calibration the peak lists were created using the ‘peak to mascot’ script of the 4700 ExplorerTM software. The resulting peak lists were analysed by using the mascot search engine (Matrix Science, London, UK), GPMAW 4.1 (Lighthouse data). The annotation of *S. aureus* NCTC8325 was used for protein identification and denotation. Peptide mixtures that yielded at least twice a Mowes score of at least 50 and a sequence coverage of at least 30% was regarded as positive identifications. Proteins that failed to exceed the 30% sequence coverage cut-off were subjected to MALDI-MS/MS (Eymann *et al*., 2004). Search parameters were as described previously (Wolf *et al*., 2008).

### 2D gel image analysis, protein quantification and statistical approaches

The 2D gel image analysis was performed with the software Delta2D version 3.6 (DECODON GmbH, Greifswald, Germany). Three different sets of biological replicates were analysed and the corresponding gel images grouped according to the genotype and oxygen level. A virtual fusion gel was created based on all autoradiograms. Spots were only detected on fusion gel and edited if necessary. This spot mask was transferred to all autoradiograms to ensure 100% spot matching. Identified spots on the fusion gel were labelled with the respective protein name. Unidentified spots were marked with a unique ID number. This consistent spot mask makes it possible to find and compare corresponding spots on each gel and in different projects. For statistical analyses, relative spot volumes were standardized (mean centre and division by standard deviation). The significance of changes in the protein synthesis of detected spots was determined by two-factorial anova for each spot considering both the genotype and the oxygen level (α = 0.01, *P*-values based on F-distribution) supported by Mev 4.1 (Saeed *et al*., 2006). Low abundance spots with a spot volume up to 0.02% on all images were eliminated from these analyses.

### Metabolic profiling of extracellular metabolites

Extracellular metabolites were detected by ^1^H-NMR as previously described (Hochgräfe *et al*., 2008). Briefly, extracellular samples were taken from the wild-type and the *rex* mutant both under aerobic and anaerobic conditions at different time points and used for NMR spectroscopy. All NMR spectra were obtained at 600.27 MHz at a nominal temperature of 298.5 K using a Bruker AVANCE-II 600 NMR spectrometer operated by TOPSPIN 2 software (both Bruker Biospin GmbH, Rheinstetten, Germany). A modified 1D-NOESY pulse sequence was used with pre-saturation on the residual HDO signal during both the relaxation delay and the mixing time. A total of 128 free induction decays (FID scans) were collected into 64 k data points using a spectral width of 20 p.p.m. for a one dimensional spectrum.
