# Supplementary material for: Redox sensing by a Rex-family repressor is involved in the regulation of anaerobic gene expression in Staphylococcus aureus
Source: Mol Microbiol. 2010 Mar 30;76(5):1142–61. doi: 10.1111/j.1365-2958.2010.07105.x (PMC2883068; doi:10.1111/j.1365-2958.2010.07105.x)
Supplement: Supplementary file 1 [file mmi0076-1142-SD1.pdf]

Table S1. Genome-wide search of potential Rex binding sites in *S. aureus* COL

Locus[a]	Symbol[a]	Description[a]	Position[b]	Putative Rex binding sites[c]	EMSA[d]	Expression under anaerobic conditions[e]		
						Gene	Operon	Protein
**3 motives without mismatches**								
SACOL0135	*(adhE)*	alcohol dehydrogenase, iron-containing	-39	T T G T G A A a t a a T T C A C A A	shift	up	up	up/down
SACOL0301	*(nirC)*	formate/nitrite transporter family protein	-51	T T G T G A A a t t a T T C A C A A	shift	up	up	
SACOL1478	*ald1*	alanine dehydrogenase	-38	T T G T G A A a t t a T T C A C A A	shift	up	up	up
**6 motives with 1 mismatch**								
SACOL0166		conserved hypothetical protein	-59	T T G T G A t t a t t T T C A C A A		up	up	
SACOL0660	*(adh1)*	alcohol dehydrogenase, zinc-containing	-306	T T G T G A A t t a a T T C A C A t	shift	up	up	up
SACOL0744		ABC transporter, ATP-binding protein, MsbA family	-2	T T G T G A A a a a a T T a A C A A				
SACOL0743	*bacA*	bacitracin resistance protein	-234	T T G T t A A t t t t T T C A C A A				
SACOL1535	*srrA*	DNA-binding response regulator SrrA	-103	T T G a G A A t t t t T T C A C A A	shift		up	up
SACOL2535	*(ddh)*	D-isomer specific 2-hydroxyacid dehydrogenase family protein	-102	T T G T G A t a t t t T T C A C A A	shift	up	up	
SACOL2534	*frp*	NAD(P)H-flavin oxidoreductase	-185	T T G T G A A a a a t a T C A C A A	shift			
SACOL2659	*aur*	zinc metalloproteinase aureolysin	-232	T T G T t A A t a t t T T C A C A A				
**9 motives with 2 mismatches**								
SACOL0079		staphylococcus tandem lipoprotein	-101	T T G T G A A a t t t T T g A t A A			up	
SACOL0204	*pflB*	formate acetyltransferase	-96	a T G T G A A a a a a a T C A C A A	shift	up	up	up
SACOL0222	*ldh1*	L-lactate dehydrogenase	-227	a T G T G A A a t a a a T C A C A A	shift			up
SACOL0220	*(hmp)*	flavohemoprotein, putative	-363	T T G T G A t t t a t T T C A C A t	shift		up	up
SACOL0222	*ldh1*	L-lactate dehydrogenase	-169	a T G T G A A a t a a a T C A C A A	shift			up
SACOL0747		cobalamin synthesis protein/P47K family protein	-158	T T G T t t A t t a a T T C A C A A				
SACOL0746	*norR*	transcriptional regulator, MarR family	-86	T T G T G A A t t a a T a a A C A A				
SACOL2007		unknown	-386	a T G T G A A t a a t a T C A C A A				

Table 1 continued

Locus[a]	Symbol[a]	Description[a]	Position[b]	Putative Rex binding sites[c]	EMSA[d]	Expression under anaerobic conditions[e] Gene	Operon	Protein
SACOL2006	*(lukM)*	aerolysin/Leukocidin family protein	-66	T T G T G A t a t t a T T C A C A t	shift	up	up	
SACOL2146	*(mtlF)*	PTS system, mannitol-specific IIBC components	-208	T T G c a A A t a t t T T C A C A A		up	up	
SACOL2364		conserved hypothetical protein	-210	a T G T G A t t t t t T T C A C A A				
SACOL2363	*(lctP)*	L-lactate permease	-124	T T G T G A A a a a a a T C A C A t	shift	up	up	
SACOL2492		hypothetical protein	-323	T T G T G A t g t a t T T C A C A A				
SACOL2491		conserved hypothetical protein	-129	T T G T G A A a t a c a T C A C A A		up	up	
**36 motives with 3 mismatches**								
SACOL0001	*dnaA*	chromosomal replication initiator protein DnaA	-398	T T a T a A A t t a t T T C c C A A				
SACOL2740	*rpmH*	ribosomal protein L34	-350	T T G g G A A a t a a T T t A t A A				
SACOL0019	*yycF (vicR)*	DNA-binding response regulator YycF	-85	c T G T G A t t t t t T a C A C A A	shift[f]			
SACOL0162		formate dehydrogenase, NAD-dependent	-252	T T G T a A A a t t a T T t A C A g			down	
SACOL0202		sensor histidine kinase family protein	-184	T a G T G c A a t a t T T a A C A A			up	
SACOL0235		hexitol dehydrogenase	-34	T T G T G A A t t a a a T C A a A t				
SACOL0319		hypothetical protein	-137	T T G g a A t t t a t T T C A C A A				
SACOL0526		DNA polymerase III, delta prime subunit, putative	-97	T T c T a A A a a t a a T C A C A A				
SACOL0552		general stress protein 13	-69	T T G T G A A a a a a T c C A a A c				
SACOLSa5SA	*5sRNA*	5S ribosomal RNA	-85	a T G T G A c a a a g T T C A C A A	no shift			
SACOL0601		hypothetical protein	-72	T g G T G A A a t t a c T C A a A A				
SACOL0602		hydrolase, haloacid dehalogenase-like family	-349	T g G T G A A a t t a c T C A a A A				
SACOL0607		azoreductase	-360	T T t a G A A a a a a T T g A C A A				down
SACOL0671		hydrolase, alpha/beta hydrolase fold family	-215	T a G T t A t a t a t T T C A C A A		up	up	
SACOL0944		NADH dehydrogenase, putative	-242	T a a T t A A t t t t T T C A C A A				

Table 1 continued

						Expression under anaerobic conditions[e]		
**Locus[a]**	**Symbol[a]**	**Description[a]**	**Position[b]**	**Putative Rex binding sites[c]**	**EMSA[d]**	**Gene**	**Operon**	**Protein**
SACOL0966	*pgi*	glucose-6-phosphate isomerase	-69	T T G T G A A t t g a a T C g C A A	no shift			
SACOL0964	*argG*	argininosuccinate synthase	-299	T T G c G A t t c a a T T C A C A A				
SACOL0965		hypothetical protein	-20	T T G c G A t t c a a T T C A C A A				
SACOL1172		hypothetical protein	-232	T T t T G A A a t t g T T C A a A A				
SACOL1188		hydrolase, haloacid dehalogenase-like family	-200	g T G T G A A a t t a g g C A C A A				
SACOL1319	*glpF*	glycerol uptake facilitator protein	-157	T T G a c A A a a t t T T t A C A A				
SACOL1318		hypothetical protein	-143	T T G T a A A a a t t T T g t C A A				
SACOL1404	*trpG*	anthranilate synthase, glutamine amidotransferase, component II	-283	T T G c G A A t t t a T T a c C A A				
SACOL1429	*asd*	aspartate-semialdehyde dehydrogenase	-52	T T G T t A A a a t a T T C t a A A				
SACOL1562		2-oxoisovalerate dehydrogenase, E1 component, alpha subunit	-378	a a G T G t A t t t a T T C A C A A				
SACOL1610	*sodA2*	superoxide dismutase	-339	T g G T G t t a a t a T T C A C A A		down	down	down
SACOL1831	*tal*	transaldolase	-39	T T G T t A A a a t t T T a A t A A				
SACOL1848		hypothetical protein	-127	T a G c c A A a a t a T T C A C A A			up	
SACOL2070	*kdpD*	sensor protein	-250	T T c T a A A a t t t T T C A C t A				
SACOL2068	*kdpA*	Potassium-transporting ATPase A chain	-39	T a G T G A A a a a t T T t A g A A				
SACOL2106		UPF0340 protein SACOL2106	-2	T T a T G A A a g a t T T g A C A A				
SACOL2112	*rpmE2*	50S ribosomal protein L31 type B	-61	T T G g G t A a a a a c T C A C A A				
SACOL2171		aerobactin biosynthesis protein, IucA/IucC family	-27	T T G T a A A a t a t T T C A t A g				
SACOL2170		transporter, putative	-92	c T a T G A A a t a t T T t A C A A				
SACOL2190		putative, uncharacterised protein	-191	T g G T G A A t t t a c T C A a A A				
SACOL2399	*nirR*	transcriptional regulator NirR	-64	a T G T G A t t c t t T T C A C A A	shift	up	up	
SACOL2409	*fmhA*	FmhA protein	-51	T T a T t A A t a a a T T C A a A A				

Table 1 continued

Locus[a]	Symbol[a]	Description[a]	Position[b]	Putative Rex binding sites[c]	EMSA[d]	Expression under anaerobic conditions[e]		
						Gene	Operon	Protein
SACOL2408		lipoprotein, putative	-212	TTtTGAA tttа TTaAtAA				
SACOL2429		putative, uncharacterised protein	-193	agGTGAA ataa TTCAaAA				
SACOL2603		putative, uncharacterised protein	-109	TTtTGAA tata aTCAtAA				
SACOL2642		putative, uncharacterised protein	-161	gTtTGAA aatt gTCACAA				
SACOL2657	*arcA*	arginine deiminase	-79	aTGTGAA tata aTCACAt	shift	up	up	

[a] A genome-wide search for potential Rex binding sites was done with the motif TTGTGAA W_4 TTCACAA against the *S. aureus* COL genome sequence. A total of 55 different motives with up to 3 mismatches up to 400 base pairs in front of the genes are shown. Locus, symbol and description of the corresponding gene are based on TIGR annotation (http://www.tigr.org). Gene symbols in brackets refer to common annotations in other *S. aureus* strains.

[b] The position of the potential Rex binding site is related to the translational start.

[c] Matches to the Rex consensus sequence are shown in capital letters. Mismatches to the Rex consensus sequence are underlined and shown in bold.

[d] Rex binding sites that have been verified by EMSA in the present study are indicated by “shift”. Motives showing no Rex binding affinity are indicated by “no shift”.

[e] Based on transcriptome and proteome data published by Fuchs *et al.* (2007).

[f] In EMSA, Rex bound to the regulatory region of *vicR* only in the presence of NAD^+ (Fig. 3).

Tab. S2 Synthetic oligonucleotides used in this study

Name	Sequence (5‘-3‘)
primers used for cloning of *rex* into pPR-IBA1	
Sa_rex_for	ATGGTAGGTCTCAAATGAGTGACCAAGTTAAAATTCCTCGAG
Sa_rex_rev	ATGGTAGGTCTCAGCGCTTTCACTGTAATTTTTCATAAAGAATAATA
primers used for Rex-DNA binding studies	
SACOL0019_for	ATAAGACGGAAAATGCGCAC
SACOL0019_rev	CTTCCATACCATCACGACCA
SACOL0301_for	ACTTAATAAATGCTCACTGCC
SACOL0301_rev	CCTCACAATGACTCCTCGC
SACOL0660_for	GACACATTTTTTTGATCATAGC
SACOL0660_rev	GCTCTCATAATAATGTCCTCC
SACOL1778_for	ATACAACTATAAATCAAATGGAG
SACOL1778_rev	AACTAACATTTGCAACACTCC
SACOL1535_for	AAATGTTGTCGGTTTGAATGC
SACOL1535_rev	TTCGACATACAGGTCATACC
SACOL2006_for	AAGATGCAGGATATTATTTAGC
SACOL2006_rev	GCACATGATAATGATGACGC
SACOL2035_for	CATTCGATCTTCACCTTTCG
SACOL2035_rev	GTCACTCATTCGCTATTTCC
SACOL2363_for	GTACAATTCATTTTGATGAACAG
SACOL2363_rev	AAACGTATTTACTAACATAGGC
SACOL1889_for	GAAAATTGGAATAGTTGATGGG
SACOL1889_rev	GTTTTTATTATGCACCATAAAGG
SACOL2399_for	ATATACACTACAAGCGACCG
SACOL2399_rev	CCATTCACATTTACCAACCC
SACOL2535_for	TATTGCTCATTGAACATAGCC
SACOL2535_rev	TTGTCATTATTAAAAACCTCGC
SACOL2563_for	ATATTGAAAATGCAATGGATCC
SACOL2563_rev	CCGTTATTCATATAACATCACC
SACOL2657_for	TATACAACGTGTTTTTGTGGG
SACOL2657_rev	CGCTATTTACTTTAATTGGACC
SACOL_Sa5SA_for	AAAATTGTATAGAATGTGTATGG
SACOL_Sa5SA _rev	ATAGCCACCAGACAAAATAAC
Sa_ldh1_for	TAAAATGTGAAATAAATCACAATTTAAT
Sa_ldh2_for	TTATATGTGAAATAAATCACAAACTTAA
Sa_adhE_for	ATATGAAACACTTAATAAAGTGTTTGTA
Sa_nirC_for	AATTTTGTGAAATTATTCACAAATAAGA
Sa_adh1_for	TATAATGTGAATTAATTCACAAAGTATA
primers used for protein-DNA interaction studies and primer extension	
SACOL0135_for	GCGATATAACAAGCTTTTTAGG
SACOL0135_rev	TCTTGTTCTTTCGATCCACG
SACOL0204_for	GTGTAACAGAATGCAATTAGC
SACOL0204_rev	TCTTCCATTTTTAAATCCTTGCC
SACOL0222_for	ATCGTATACAAATTAAAAAGGTG
SACOL0222_rev	TTTGTTCATTACAAAAACTCCC
primers used for cloning the *adhE* upstream region	
SACOL0135_for2	CATTTTTGAATCATCTAGCAGG
SACOL0135_rev2	ATATGTTGATCAACAGCTGC
primers used for RNA-probe synthesis	
*rex*_for	TGAGTGACCAAGTTAAAATTCC
*rex*_rev[1]	CTAATACGACTCACTATAGGGAGATAATAATGACTGTAATTCTATACC
*ldh1*_for	CATGCCACACCATATTCTCC

*ldh1*_rev[1]	CTAATACGACTCACTATAGGGAGAGCTGATACAGTCAATACGGC
*lctP*_for	GCAGAATGTGCTTTTGCAGG
*lctP*_ rev[1]	CTAATACGACTCACTATAGGGAGAGCAGAATGTGCTTTTGCAGG
*srrA*_for	CCATGAAGCAAGTAATGGCC
*lctP*_ rev[1]	CTAATACGACTCACTATAGGGAGATCAAATTTATACCCAACGCCC
*adh1*_for	GTCTATCGCTTGGATGTTCG
*adh1*_ rev[1]	TAATACGACTCACTATAGGGAGACCGTCAAGCACTAATCTTGG
*adhE*_for	ATGCTCTAGCTGACAAAGGG
*adhE*_ rev[1]	CTAATACGACTCACTATAGGGATGTGCACTTGGATGGAATGC
*ald1*_for	TTGCACACCCGAAAATGTGC
*ald1*_rev[1]	CTAATACGACTCACTATAGGGAGATGCGGTCATCGTTTAACTCG
*pflB*_for	GGTGCAGCAATGAGTTTAGG
*pflB*_ rev[1]	CTAATACGACTCACTATAGGGAGATTTGGACCAACTTGTGCACC
*nirR*_for	TTGTTGCACATGGCATGAGG
*nirR*_ rev[1]	CTAATACGACTCACTATAGGGAGAACCATGTCATTCATTTGCTGC
*narH*_for	TACCACGTTTATGTGAACATTGC
*narH*_ rev[1]	CTAATACGACTCACTATAGGGAGATCGGCATCGTTCTAAATTCAGG
*clpL*_for	AAAAATAACACACAATATTC
*clpL*_ rev[1]	CTAATACGACTCACTATAGGGAGACTCAACCGATAATTTGATGG
*vicR*_for	GTGTACTGTGCATACGATGG
*vicR*_ rev[1]	CTAATACGACTCACTATAGGGAGAAATATCCAACGCCTCTACGC

[1] underlined sequence corresponds to the T7 polymerase binding site (Jorgensen et al., 1991)

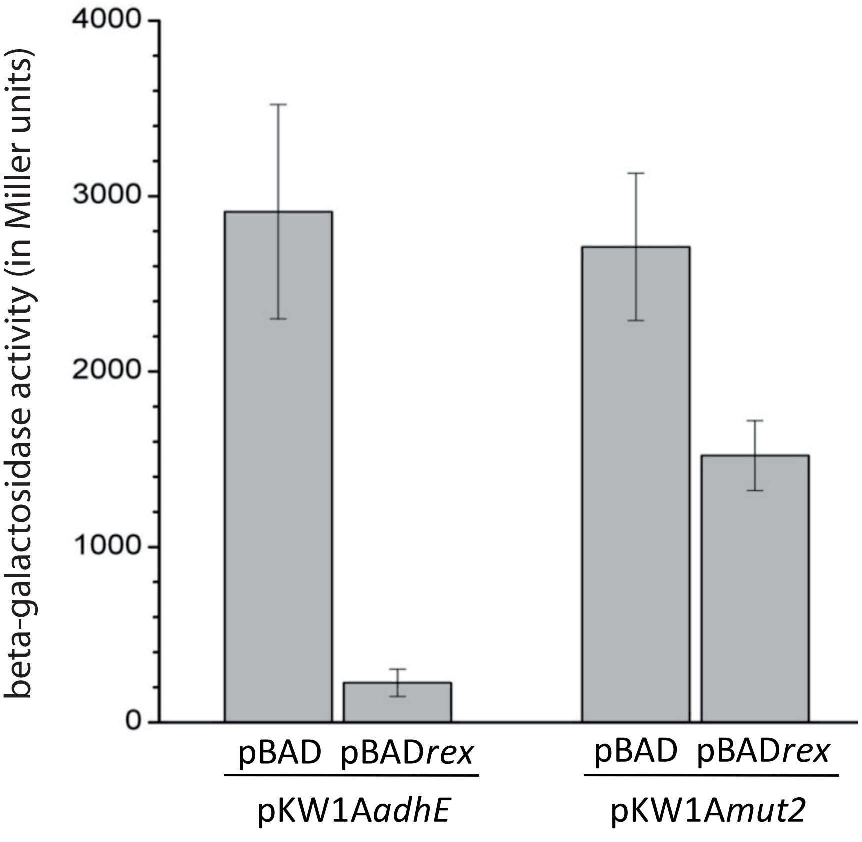

beta-galactosidase activity (in Miller units)
4000
3000
2000
1000
0
pBAD
pBADrex
pBAD
pBADrex
pKW1AadhE
pKW1Amut2

Figure S1

Legend to Fig. 1S

Characterisation of the *in vivo* effect of Rex binding to the a*dhE* upstream region by β-galactosidase assays

Escherichia coli DH5α was transformed with plasmid pKW1A*adhE* (transcriptional fusion of the *adhE* upstream region of *S. aureus* to the *lacZYA* operon of *E. coli*) or pKW1A*mut2* (transcriptional fusion of the *adhE* upstream region of *S. aureus* with the mutated Rex binding site (mutant 2) to the *lacZYA* operon of *E. coli*). In a second step, verified clones were either transformed with plasmid pBAD*rex* (carrying the *rex* gene of *S. aureus* COL) or pBAD (Guzman *et al.,* 1995) as a control. β-galactosidase activity is shown as average value of Miller units of three biological replicates with the respective standard deviation.

Briefly, overnight cultures were grown at 37°C and 160 rpm in LB medium, 1:20 diluted in fresh medium containing 2% arabinose and incubated for 2 h under mentioned conditions. The cultures were then collected by centrifugation at 4°C and washed in cold 0.85% (w/v) NaCl before enzyme activity assays were performed. β-galactosidase enzyme activities are expressed in arbitrary units, which were determined according to the formula of Miller *et al.* (1972). Cultures were assayed in triplicate and reported values are averaged from at least four different experiments. Bacteria carrying the reporter plasmid containing the *adhE* promoter but empty expression plasmids served as controls.

References

Guzman L.M., Belin D., Carson M.J., and Beckwith J. (1995) Tight regulation, modulation, and high-level expression by vectors containing the arabinose PBAD promoter. *J Bacteriol* **177**: 4121-4130.

Miller J.H. (1972) Experiments in molecular genetics. Cold Spring Harbor Laboratory Press, Cold Spring Harbor, NY

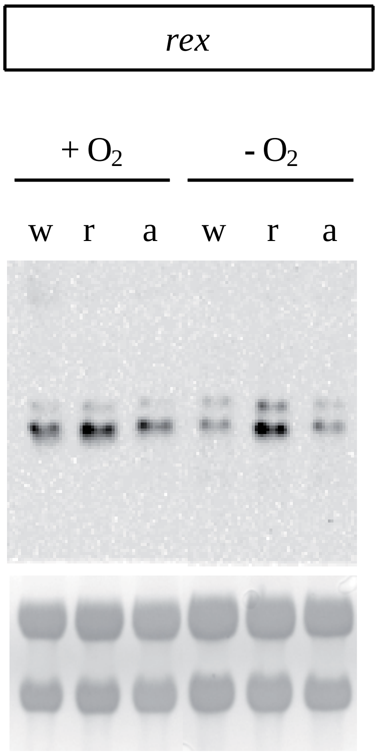

Figure S2

A

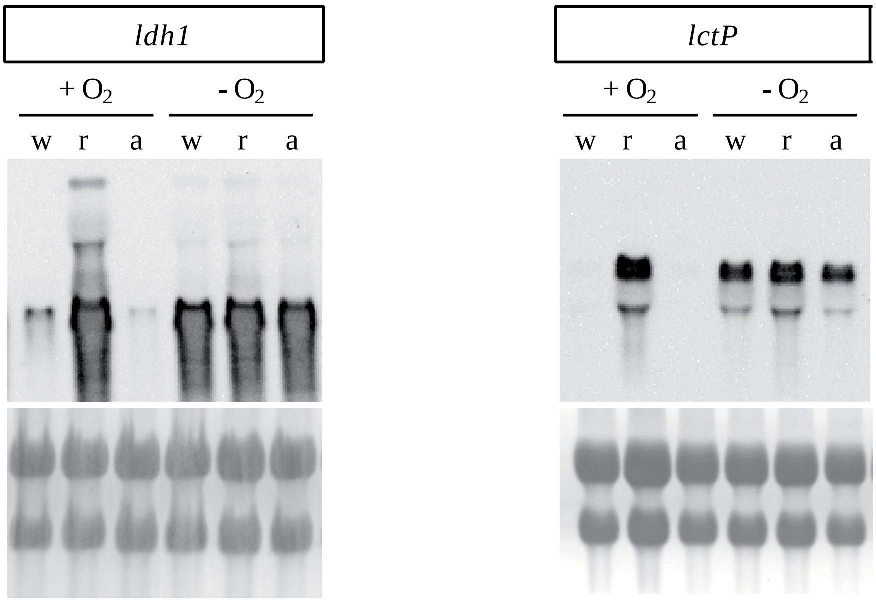
ldh1
+ O2
- O2
w r a w r a
lctP
+ O2
- O2
w r a w r a

B

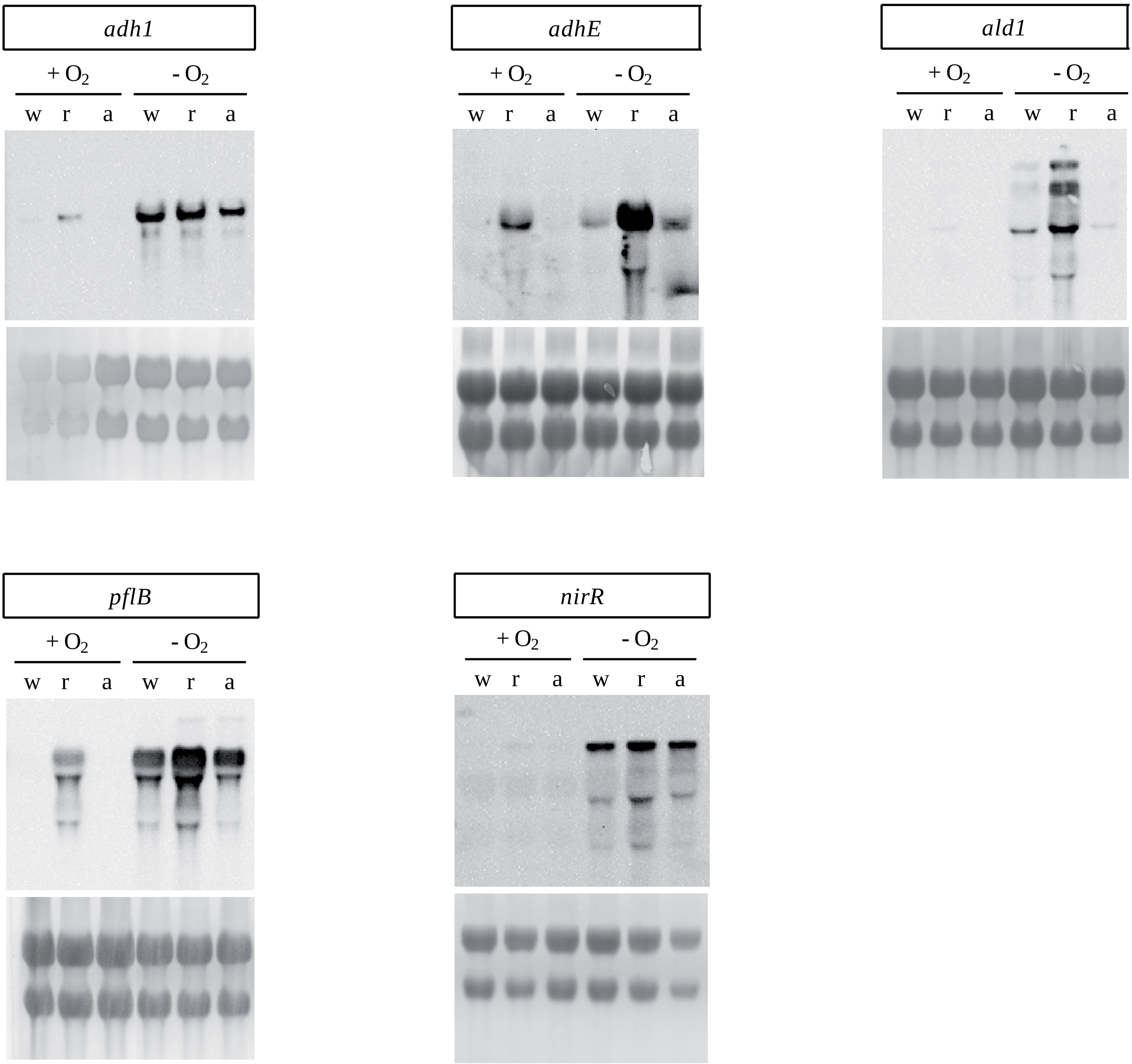
adh1
+ O2
- O2
w r a w r a
adhE
+ O2
- O2
w r a w r a
ald1
+ O2
- O2
w r a w r a
pflB
+ O2
- O2
w r a w r a
nirR
+ O2
- O2
w r a w r a

C

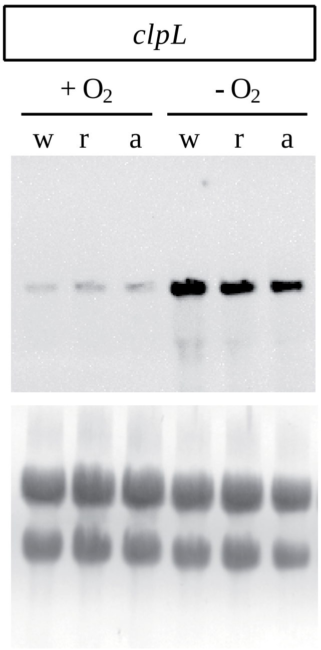

Figure S3
